# A Sugarcane G-Protein-Coupled Receptor, *ShGPCR1*, Confers Tolerance to Multiple Abiotic Stresses

**DOI:** 10.3389/fpls.2021.745891

**Published:** 2021-11-11

**Authors:** Manikandan Ramasamy, Mona B. Damaj, Carol Vargas-Bautista, Victoria Mora, Jiaxing Liu, Carmen S. Padilla, Sonia Irigoyen, Tripti Saini, Nirakar Sahoo, Jorge A. DaSilva, Kranthi K. Mandadi

**Affiliations:** ^1^Texas A&M AgriLife Research and Extension Center, Weslaco, TX, United States; ^2^Department of Biology, University of Texas Rio Grande Valley, Edinburg, TX, United States; ^3^Department of Soil and Crop Sciences, Texas A&M University, College Station, TX, United States; ^4^Department of Plant Pathology and Microbiology, Texas A&M University, College Station, TX, United States

**Keywords:** abiotic stresses, G-protein-coupled receptor, sugarcane, transgenics, bioenergy and biofuel

## Abstract

Sugarcane (*Saccharum* spp.) is a prominent source of sugar and serves as bioenergy/biomass feedstock globally. Multiple biotic and abiotic stresses, including drought, salinity, and cold, adversely affect sugarcane yield. G-protein-coupled receptors (GPCRs) are components of G-protein-mediated signaling affecting plant growth, development, and stress responses. Here, we identified a GPCR-like protein (*ShGPCR1*) from sugarcane and energy cane (*Saccharum* spp. hybrids) and characterized its function in conferring tolerance to multiple abiotic stresses. *ShGPCR1* protein sequence contained nine predicted transmembrane (TM) domains connected by four extracellular and four intracellular loops, which could interact with various ligands and heterotrimeric G proteins in the cells. *ShGPCR1* sequence displayed other signature features of a GPCR, such as a putative guanidine triphosphate (GTP)-binding domain, as well as multiple myristoylation and protein phosphorylation sites, presumably important for its biochemical function. Expression of *ShGPCR1* was upregulated by drought, salinity, and cold stresses. Subcellular imaging and calcium (Ca^2+^) measurements revealed that *ShGPCR1* predominantly localized to the plasma membrane and enhanced intracellular Ca^2+^ levels in response to GTP, respectively. Furthermore, constitutive overexpression of *ShGPCR1* in sugarcane conferred tolerance to the three stressors. The stress-tolerance phenotype of the transgenic lines corresponded with activation of multiple drought-, salinity-, and cold-stress marker genes, such as *Saccharum* spp. *LATE EMBRYOGENESIS ABUNDANT*, *DEHYDRIN*, *DROUGHT RESPONSIVE 4*, *GALACTINOL SYNTHASE, ETHYLENE RESPONSIVE FACTOR 3*, *SALT OVERLY SENSITIVE 1*, *VACUOLAR Na+/H+ ANTIPORTER 1*, *NAM*/*ATAF1/2*/*CUC2*, *COLD RESPONSIVE FACTOR* 2, and *ALCOHOL DEHYDROGENASE 3*. We suggest that *ShGPCR1* plays a key role in conferring tolerance to multiple abiotic stresses, and the engineered lines may be useful to enhance sugarcane production in marginal environments with fewer resources.

## Introduction

Membrane-localized receptors play key roles in signal perception and transduction in downstream intra- and intercellular signaling networks (Khatri et al., 2012). Among them, G-protein-coupled receptors (GPCRs) are a conserved family of membrane-bound proteins present in most eukaryotes (Jacoby et al., 2006; Tuteja, 2009; Trusov and Botella, 2016). GPCRs mediate responses to several physiological processes, such as growth, development, and extracellular stimuli. Members of the GPCR protein family share a common central core domain composed of 7–9 transmembrane (TM) helices connected by three N-terminal extracellular loops and three C-terminal intracellular loops, a distinct characteristic not seen in other classes of cell membrane receptors (Ding et al., 2013; Ofoe, 2021). Studies of the few plant GPCRs characterized to date (Pandey and Assmann, 2004; Liu et al., 2007; Pandey et al., 2009; Ma et al., 2015) have provided evidence that plants use similar mechanisms to other eukaryotes to regulate G-protein-mediated signaling, although the signal inputs are different. In plants, GPCRs are involved in diverse abiotic stress responses. For instance, loss-of-function mutants in distinct *GPCR* genes in *Arabidopsis* (*Arabidopsis thaliana*) show hypersensitivity to abscisic acid (ABA) and drought (Chen et al., 2006), salt (Chakravorty et al., 2015), and oxidative stress (Joo et al., 2005). The role of GPCRs in crop plants, such as rice (*Oryza sativa*) and maize (*Zea mays*), has also been investigated (Ma et al., 2015; Ferrero-Serrano and Assmann, 2016).

At the biochemical level, intracellular events of the signaling cascade are initiated when GPCRs bind to signal molecules (ligands) and undergo conformational changes. Upon ligand binding, the activated receptor promotes the disassociation of the G-protein *α*-subunit from the *βγ*-subunit heterodimer complex and facilitates the exchange of guanidine triphosphate (GTP) for guanidine diphosphate (GDP) on the G-protein *α*-subunit (Ross and Wilkie, 2000; Oldham and Hamm, 2008). Both activated parts remain attached to the plasma membrane but are now free to induce specific responses through their respective downstream signaling effectors (Temple and Jones, 2007; Pandey et al., 2009; Khatri et al., 2012; Sprang, 2016). The G*α*-subunit remains in its active form for only a limited time, due to an intrinsic GTPase activity deployed for deactivation. Once the G*α*-subunit hydrolyzes GTP to GDP, the heterotrimer reforms, and signaling is terminated. Interestingly, some plant G*α* proteins can self-activate and do not always require a GPCR to relay signals (Xu et al., 2016). Furthermore, signal transduction can be modulated by phosphorylation of GPCRs and G-protein complexes by single-transmembrane receptor kinases that act as primary regulators (Jia et al., 2019).

Plants possess a limited number of GPCRs and G-protein components compared to humans or other organisms. While approximately 4% of the human genome is encoded by GPCRs (more than 1,000 human GPCRs; Alexander et al., 2019), only 50 proteins have been identified in Arabidopsis and rice that potentially possess the same topology as human GPCRs (Gookin et al., 2008; Pandey and Vijayakumar, 2018). The best characterized plant GPCR-like proteins are the Arabidopsis GPCR-type G proteins GTG1 and GTG2, which bind to the phytohormone ABA preferentially in their GDP-bound form (Pandey et al., 2009); the GPCR 1 protein GCR1, whose ligand is unknown (Pandey and Assmann, 2004); and the ABA receptor GCR2 (Liu et al., 2007; Shi and Yang, 2015). Other GPCR-like proteins have been identified in crops, including rice CHILLING-TOLERANCE DIVERGENCE 1 (COLD1; Ma et al., 2015), *Os*GPCR (Yadav and Tuteja, 2011), cotton (*Gossypium hirsutum*) target of Myb1 (*Gh*TOM1; Lu et al., 2018, 2019), and pea (*Pisum sativum*) GPCR (Misra et al., 2007). In Arabidopsis, GTG1 and GTG2 are associated with enhanced seedling growth and fertility (Jaffé et al., 2012), seed germination and root elongation (Pandey et al., 2006), and organ differentiation (Ullah et al., 2003) as well as light signal transduction (Warpeha et al., 2006). GPCRs from Arabidopsis and rice appear to also mediate changes in cellular calcium (Ca^2+^) levels (Ma et al., 2015) and ABA signaling (Wang et al., 2001; Assmann, 2002; Pandey et al., 2006, 2008, 2009; Misra et al., 2007). The identity and function of GPCRs are other high-value agronomic crops like sugarcane and energy cane (*Saccharum* spp. hybrids), which are prominent sources of sugar-based ethanol and lignocellulosic biomass feedstocks globally remain less explored.

Multiple biotic and abiotic stresses, including drought, salinity, and cold, adversely affect sugarcane growth and result in yield losses of 50–60% (Basnayake et al., 2012; Gentile et al., 2015; Park et al., 2015; Da Silva, 2017; Ali et al., 2019). Among them, drought is one of the most limiting factors in sugarcane production, with water deficit leading to yield losses of 50–60% (Basnayake et al., 2012; Gentile et al., 2015; Da Silva, 2017). Drought, salinity, and cold stresses in sugarcane typically result in leaf rolling, stomatal closure, inhibition of culm and leaf growth, and leaf chlorosis, necrosis, and senescence (Inman-Bamber and Smith, 2005; Inman-Bamber et al., 2012). Root development is also affected by these stresses (Smit and Singels, 2006), but to a lesser degree than aboveground biomass. One way to enhance sugarcane stress tolerance to abiotic stresses is by manipulating GPCR activity. Unfortunately, lack of a high-quality reference genome and the relative recalcitrance to genetic transformation makes sugarcane a challenging system for genetic studies and crop improvement. Sugarcane interspecific hybrids have also large polyploid genomes (~10Gbp) and are highly complex, with varying chromosome numbers (Grivet and Arruda, 2002; Vermerris, 2011; De Setta et al., 2014). However, a recently released draft genome of the autopolyploid sugarcane *S. spontaneum* L. provides some resources to accelerate sugarcane improvement (Garsmeur et al., 2018; Zhang et al., 2018). In this study, we report the identification, isolation, and functional characterization of a *GPCR* gene from *Saccharum* spp. hybrids (*ShGPCR1*) and show that genetically modified sugarcane plants overexpressing *ShGPCR1* are more resistant to drought, salinity, and cold stresses.

## Materials and Methods

### Identification of *ShGPCR1*

The protein coding sequence (CDS) of an orthologs *GPCR* gene from rice (LOC_Os04g51180.1) was retrieved and used to perform a Basic Local Alignment Tool (BLAST) search (*E*-value<1e-05) against the sugarcane expressed sequence tag (SUCEST) database (Vettore et al., 2003). SUCEST is a comprehensive collection of sugarcane RNA-sequencing (RNA-seq) transcript assemblies, with 237,954 high-quality ESTs prepared from 26 diverse tissue-specific cDNA libraries from 13 commercial sugarcane varieties (Vettore et al., 2001). Additionally, we mined publicly available sugarcane RNA-seq datasets through the National Center for Biotechnology Information (NCBI) Short Read Archive BLAST tool (Misra et al., 2007). We used all recovered reads (NCBI-SRA) and transcript hits (SUCEST) to assemble a consensus *in silico* sequence sharing highest similarity to the rice *GPCR*. The consensus transcript assembly defined a CDS of 1,407 nucleotides and encoded a GPCR-like protein. During the preparation stages of this manuscript, an allele-defined sugarcane draft genome was released (Garsmeur et al., 2018; Zhang et al., 2018). We compared our predicted *ShGPCR1* sequence to the draft sugarcane genome, and found a single, perfectly matching locus.

### Cloning and Sequencing of *ShGPCR1* Alleles

We isolated total RNA from 100-mg sugarcane (variety CP72-1210) and energy cane (variety TCP10-4928) leaves using the Direct-zol RNA MicroPrep kit (Zymo Research, Irvine, CA, United States). We synthesized first-strand cDNAs for reverse transcription PCR (RT-PCR) using 1-μg total RNA and SuperScript III Reverse Transcriptase (Thermo Fisher Scientific, Waltham, MA, United States) with oligo(dT)_20_ primer according to the manufacturer’s instructions. We amplified the *GPCR*-like sequence *ShGPCR1* from sugarcane and energy cane using primers that recognize the start and stop codons of the gene, *ShGPCR1-F* (5'-GCGAGGAATACAGCAAGGGA-3') and *ShGPCR1-R* (5'-TGGGTCACCAAAGAAACATC-3'). We performed PCR reactions on a ProFlex™ PCR System (Applied Biosystems by Life Technologies, Carlsbad, CA, United States) in a total reaction volume of 50μl using 1μl of cDNA, 0.5μM of each target-specific primer, and 1.0U of Phusion DNA polymerase (New England BioLabs, Ipswich, MA, United States). PCR conditions were as: one denaturing cycle at 98°C for 30s, 30cycles each at 98°C for 15s, 62°C for 15s, and 72°C for 2min, and a final extension cycle at 72°C for 5min. We separated PCR amplicons from sugarcane and energy cane by electrophoresis on a 1% (w/v) agarose gel, before cloning into the pTEM73 vector and transformation into *Escherichia coli* strain DH5α. We isolated plasmid DNA from 10 randomly selected recombinant colonies using the Plasmid MiniPrep Kit (Zymo Research, Irvine, CA, United States) and by Sanger DNA-sequencing determined the identify of *ShGPCR1* alleles present in sugarcane and energy cane (Table 1; Supplementary Dataset).

**Table 1:** Haplotype analysis of *ShGPCR* alleles in sugarcane and energy cane.

Sequence ID	CDS length (nt)	Protein length (aa)	Single nucleotide polymorphisms (SNPs)	Non-synonymous SNPs	Amino acid changes
*In silico* sequence	1,407	468	-	-	-
EC1	1,407	468	G49C, G107A, C238G, G318A, G789A, and T1164C	G49C, G107A, and C238G	Val17Leu, Cys36Tyr, and Leu80Val
EC2	1,407	468	G49C, G107A	G49C, G107A	Val17Leu, Cys36Tyr
EC3	1,407	468	G49C, G107A, C238G, G318A, G693A, G855A, and T1164C	G49C, G107A, and C238G	Val17Leu, Cys36Tyr, and Leu80Val
EC4 (*ShGPCR1*)	1,407	468	G49C, G107A, G789A, and T1164C	G49C, G107A	Val17Leu, Cys36Tyr
EC5	1,407	468	G48C, G49C, G107A, C831A, T1134C, G1158A, and T1164C	G49C, G107A	Val17Leu, Cys36Tyr
SC1	1,407	468	G48C, G49C, G107A, C831A, and T1034C	G49C, G107A, and T1034C	Val17Leu, Cys36Tyr, and Val345Ala
SC2	981	325	G49C, G107A, and Δ964-967	G49C, G107A	Val17Leu, Cys36Tyr
SC3	1,407	468	G49C, G107A, T445C, G853T, T1134C, and G1158A	G49C, G107A, and G853T	Val17Leu, Cys36Tyr, and Ala285Ser
SC4	1,407	468	G48C, G49C, G107A, C831A, and T1034C	G49C, G107A, and T1034C	Val17Leu, Cys36Tyr, and Val345Ala
SC5	981	325	G49C, G107A, and Δ962-965	G49C, G107A	Val17Leu, Cys36Tyr

EC, energy cane; SC, sugarcane; nt, nucleotide; aa, amino acid; and Δ, mutation.

### *In silico* Analysis of *ShGPCR1*

We used the DNA sequence of *GPCR* genes to deduce their amino acid sequences using the translate tool at the ExPaSy Bioinformatics Resource Portal.^1^ We performed a homology search for the deduced amino acid sequences of *ShGPCR1* from sugarcane and energy cane, using NCBI BLAST.^2^ We then compared the amino acid sequence of the selected *ShGPCR1* with GPCR-like proteins from Arabidopsis, rice, sorghum, maize, and cotton by multiple amino acid sequence alignment using ClustalW2.0.^3^ We performed phylogenetic analyses using the Neighbor-Joining method with pair-wise deletion of alignment gaps and Poisson correction for amino acid substitutions in MEGAX (Kumar et al., 2018). We used the ExPaSy PROSITE database of protein families and domains (Sigrist et al., 2013) to identify functional motifs and biologically significant sites in *ShGPCR1*. We predicted the presence of transmembrane domains using the Trans Membrane Hidden Markov Model 2 (TMHMM2) program (Krogh et al., 2001).

### Subcellular Localization of *ShGPCR1*

We generated embryogenic leaf rolls of sugarcane (variety CP72-1210) and subjected them to DNA bombardment as described above, with the following modifications. Embryogenic leaf rolls were cultured on MS0.6 medium (MS medium supplemented with 0.6mg/l of 2,4-D) for 8days in the dark at 28°C. Leaf roll disks (1mm diameter; 2–3 disks per bombardment) were preconditioned on MS0.6-osmoticum (MS6 with 0.2M D-mannitol and 0.2M D-sorbitol) for 4h before DNA particle bombardment. Gold particles were coated separately with plasmid DNA (5.0μg) of pTEM73-*ShGPCR1-mGFP* or pTEM73-*mGFP*. Twenty four hours after bombardment and incubation on MS0.6-osmoticum, leaf roll disks were transferred into MS3 medium and kept in the dark at 28°C for 10days prior to imaging. We rinsed the disks three times with water and sectioned them into 1mm thick pieces for microscopy; the pieces were stained with the cell membrane-specific lipophilic dye FM4-64 (Invitrogen, Thermo Fisher Scientific) for plasma membrane visualization (Lam et al., 2008), or with DAPI (Invitrogen, Thermo Fisher Scientific) for staining of nuclei. Stained pieces were rinsed three times with water and imaged on a Fluoview FV10i-LIV confocal laser scanning microscope (Olympus Life Sciences, Waltham, MA, United States) at 488, 546, and 385-nm excitation wavelengths for GFP, FM4-64, and DAPI, respectively. Images were acquired using the *Z*-stack scan mode (acquisition of images in different focus positions). Ten image planes were scanned with the optimal pixel size of 0.1μm.

### Construction of Expression Vectors

The *ShGPCR1* coding region (1,407bp) was PCR amplified from the cloned *ShGPCR1* cDNA using gene-specific primers (Supplementary Table S1) and cloned at the *Bam*HI and *Pml*I sites, replacing the custom synthesized codon-optimized (GenScript, Piscataway, NJ, United States) bialaphos-resistance (*bar*) gene into the minimal plant expression vector pTEM73 (Beyene et al., 2011) under control of the maize *Ubi* promoter (including first exon and intron) and the *Cauliflower mosaic virus* (CaMV) *35STTnos* double terminator to generate pTEM73-*ShGPCR1*. The pTEM73-*ShGPCR1* and pTEM73 (containing the *bar* selectable marker) were further used to perform biolistic-based co-transformation of sugarcane (Bower et al., 1996; Ramasamy et al., 2018). For subcellular localization of *ShGPCR1*, we PCR amplified the open reading frame of the mutant Green Fluorescent Protein *mGFP* (Haseloff et al., 1997) using high fidelity Phusion DNA polymerase (New England BioLabs, Ipswich, MA, United States). We cloned the *mGFP* PCR product by Gibson assembly (Gibson et al., 2009) into the pTEM73 vector downstream of the *ShGPCR1* CDS, producing the pTEM73-*ShGPCR1-mGFP* construct, in which the chimeric gene is driven by the *Ubi* promoter and *35STTnos* double terminator. Similarly, we cloned *mGFP* into pTEM73 to generate the control vector pTEM73-*mGFP*.

### Sugarcane Transformation and Selection of Transgenics

We collected tops of field-grown sugarcane (*Saccharum* spp. hybrids), commercial variety CP72-1210, during the growing season, and prepared leaf rolls for transformation, as previously described (Gao et al., 2013; Ramasamy et al., 2018). Briefly, we surface-sterilized immature leaf rolls close to the apical meristem in 70% (v/v) ethanol for 20min, sliced them transversely into 1mm thick sections, and cultured them on MS (Murashige and Skoog, 1962) supplemented with 3mg/l of 2,4-dichlorophenoxyacetic acid [2,4-D] (MS3 medium) for 30–35days at 28°C. Embryogenic calli were preconditioned on MS3-osmoticum (MS3 with 0.2M D-mannitol and 0.2M D-sorbitol) for 4h before and after DNA particle bombardment, which was performed according to Ramasamy et al. (2018). Briefly, for DNA particle coating, we added 5.0μg of each of pTEM73-*ShGPCR1* and pTEM73 (*bar* selectable marker) plasmids sequentially to 5.0-μg gold particles (0.3μm, Crescent chemical Co, NY, United States) suspension using 1M-calcium chloride and 14-mM spermidine. Next, we placed 4μl of this DNA particle suspension (0.5μg DNA/bombardment) at the center of a syringe filter and delivered into tissue with a particle inflow gun using a 26-in Hg vacuum and 7-cm-target distances. We incubated bombarded embryogenic calli on MS3 medium for 10–12days in the dark for recovery. Shoot regeneration was performed under selection on MS medium with 1.5mg/l of benzylaminopurine and 3mg/l of bialaphos, followed by root initiation on MS medium with 4mg/l of indolebutyric acid and 3mg/l of bialaphos. After 6–8weeks, once root formation was well established, we transplanted transgenic seedlings into potting soil (Sunshine Mix #1; Sun Gro Horticulture, Belleview, WA, United States) and moved them to a controlled-environment greenhouse.

We verified the presence of the *ShGPCR1* and *bar* (selectable marker) genes in co-transformed sugarcane plants by PCR using the forward primer (5'-GATGCTCACCCTGTTGTTTG-3') from the *Ubi* promoter and the reverse primer (5'-GACAGATCGAGCTCTGACTAGG-3') from the *Tnos* terminator. We performed PCR on a ProFlex™ PCR System in a total reaction volume of 25μl using 100ng of genomic DNA (isolated from 0.5–1g of young leaves of 3–4-month-old plants using the protocol of Chiong et al., 2017), 0.1μM of each target-specific primer, and 1.0U of *Taq* DNA polymerase and ThermoPol™ buffer (New England BioLabs, Ipswich, MA, United States). PCR conditions were as: one denaturing cycle at 95°C for 30s, 30cycles each at 95°C for 15s, 52°C for 15s, and 68°C for 1min, and a final extension cycle at 68°C for 5min. The amplified PCR products were ~1,640 and 500bp in size, respectively. Plants with the expected size amplicons were selected for further molecular analysis.

Integration of the *ShGPCR1* expression cassette into the sugarcane genome was determined by Southern blot hybridization. We digested genomic DNA (10μg/reaction) overnight with *Hind*III, electrophoresed on 0.8% (w/v) agarose gels, and transferred into nylon membranes (Amersham Hybond-XL, GE Healthcare Bio-Sciences Corp., Piscataway, NJ) in 0.4-M sodium hydroxide (Koetsier et al., 1993). Pre-hybridization, hybridization, washing, and detection of DNA gel blots were performed, using Church’s buffer as described by Sambrook et al. (1989) and Mangwende et al. (2009). The *ShGPCR1*-specific probe (1,407bp) was released from pTEM73-*ShGPCR1* with *Hind*III digest and labeled with [α-^32^P]dCTP using the Random Primers DNA Labeling kit (Invitrogen, ThermoFisher Scientific, Waltham, MA, United States).

### Propagation and Abiotic Stress Assays

We propagated sugarcane seedlings *in vitro* on half-strength MS medium, transplanted them later to Sunshine Mix #1 (Sun Gro Horticulture, Agawam, MA, United States) in plastic pots, and moved them to a controlled-environment greenhouse (25–30°C during the day and 15–24°C at night; 1,200–1,600μmol/m^2^/s at midday). Plants were fertilized once a week with soluble Peters® Professional 20-20-20 (The Scotts Company, Marysville, OH; 1.6g/l). For stress-inducible expression of endogenous *ShGPCR1* in sugarcane (variety CP72-1210), we moved 10-week-old seedlings grown in the greenhouse to a 28°C growth chamber for a 1-week acclimation period before subjecting them to cold, drought, or salinity. For cold treatment, seedlings were moved from a 28°C growth chamber to one maintained at 0°C. For drought stress, seedlings were carefully pulled out of potting medium and left to wilt on a tray for 2, 6, and 28h. For salinity treatment, we drenched the soil with 200-mM NaCl. For ABA treatment, detached leaves (4cm in length) were treated with 10, 25, 50, and 100nM of ABA for 10h. Samples were collected from each of the stress-treated and untreated control seedlings at 2, 6, and 28h post-treatment, flash-frozen in liquid nitrogen, and stored at −80°C. For *ShGPCR1* expression analysis, we pooled equal amounts of total RNA extracted from tissue at each of the three time points.

We also analyzed the expression of *ShGPCR1* in three *ShGPCR1*:*OE* lines under drought, salinity, and cold stresses (Begcy et al., 2012; Belintani et al., 2012; Da Silva, 2017). We performed drought stress-tolerance assays with 4-month-old *ShGPCR1-OE* and NT plants derived from tissue culture, acclimated in well-watered conditions in 15-L plastic pots for 1week in a controlled-environment greenhouse and later subjected to a progressive drought by withholding water for 40days until soil moisture reached ~10% for NT and~15% for transgenic plants. For salinity stress-tolerance assays, we watered 1-month-old *ShGPCR1-OE* and NT plants with 200-mM NaCl (200ml per 1-L pot) in reverse-osmosis water by soil drenching once a week for a period of 2weeks. For chilling stress-tolerance assays, we treated 2-month-old *ShGPCR1:OE* and NT plants grown in the greenhouse at 4°C for 3weeks, followed by −5°C for 4h in a temperature-controlled growth chamber. We also performed chilling treatments with 2-week-old seedlings in MS medium in Magenta boxes at 4°C for 24h, followed by −20°C for 4h. Foliar symptoms of stress were evaluated, and severity of symptoms was rated based on a scale of 1–3, where 1=mild (few leaf curling and wilting), 2=moderate (<25–50% of leaves showing curling and wilting with concomitant necrosis), and 3=severe (>75% of leaves showing leaf curling and wilting with concomitant necrosis). Leaf tissues of control and stressed *ShGPCR1-OE* and NT plants were harvested, flash-frozen in liquid nitrogen, and kept at −80°C for expression analysis of *ShGPCR1* and stress-related marker genes (*LEA*, *DHY*, *SCDR4*, and *GOLS* for drought, *ERF3*, *SOS1*, and *ShNHX1* for salinity, and *SsNAC23*, *CBF2*, and *ScADH3* for cold; Supplementary Table S1). All stress experiments were carried out in a randomized block design using 3–4 biological replications, i.e., independent plants per line per treatment.

### Measurement of Relative Water Content and Agronomic Parameters

We evaluated the *ShGPCR1-OE* lines and NT plants for leaf relative water content (RWC) and agronomic parameters every 7days for a period of 40days of progressive drought stress. We measured RWC using the detached leaf method at the end of the drought stress treatment (Dhanda and Sethi, 1998). We cut fully expanded leaves (1cm×4cm), weighed them to obtain fresh weight (FW), floated them on de-ionized water in a Petri dish, and kept at 10°C for 4h. We then patted the resulting turgid leaves dry on filter paper to remove excess water and weighed them again to obtain their turgid weight. After weighing, we dried the leaf segments at 80°C in a hot air oven for 24h before measuring their dry weight. We calculated RWC using the formula: RWC (%)=[(FW−DW)/(TW−DW)]×100. The agronomic parameters considered and measured here were culm diameter (cm), culm height (cm), flag leaf length (cm), and dry root weight (g). We also estimated total biomass (aboveground parts, such as culms and leaves) by calculating the fresh biomass weight and DW after drying of samples at 80°C until they reached a constant weight, using an analytical balance. We calculated the total biomass using the formula: Total biomass (%)=(DW of total biomass/FW of total biomass)×100. Significant differences were determined using two-sample Student’s *t*-test (*p*<0.05).

### RNA Isolation and Gene Expression Analyses

To measure *ShGPCR1* transcript levels in sugarcane, we isolated total RNA from 0.5g young leaves of stress-treated wild type and corresponding untreated controls, using the Direct-zol™ RNA MiniPrep kit (Zymo Research, Irvine, CA, United States). We synthesized first-strand cDNAs using 1-μg total RNA and the SuperScript™ IV Reverse transcription kit (Invitrogen, ThermoFisher Scientific). We performed quantitative RT-PCR (RT-qPCR) on a CFX384 Real-Time System (Bio-Rad Laboratories, Inc., Hercules, CA, United States) with the i*Taq*™ Universal SYBR Green Supermix (Bio-Rad Laboratories, Inc.) using 3–4 biological replicate samples and two technical replicates for qPCR analysis. Primers were designed with Primer 3.0.^4^ Results were analyzed and recorded as C_T_ (threshold cycle) values. We quantified each transcript relative to the sugarcane reference gene, *ANTHRANILATE PHOSPHORIBOSYLTRANSFERASE* (*APRT*, GenBank CA089592.1; Casu et al., 2012), using the comparative C_T_ method (2^−ΔΔC^_T_). Primer pairs used for RT-qPCR were as: *ShGPCR1-F2* (5''-AAGTCCAGGCACTGGAAGAG-3'') and *ShGPCR1-R2* (5''-AACACAATACACCGACAAAGCA-3''), and *APRT2-F* (5'-CGGTCGTTTCTGGTTTTGTT-3') and *APRT2-R* (5'-CGCCAAGAATGTGGTATGTG-3'). Significant differences were determined using two-sample Student’s *t*-test (*p*<0.05).

To examine the accumulation of *ShGPCR1* transcripts in the sugarcane *ShGPCR1-OE* lines, we performed RT-PCR on a ProFlex PCR System in a total reaction volume of 25μl using 1μl of cDNA (synthesized from 1μg of total RNA extracted from leaves), 0.1μM of each target-specific primer, and 1.0U of *Taq* DNA polymerase and ThermoPol™ buffer (New England BioLabs). PCR conditions were as: one denaturing cycle at 95°C for 30s, 30cycles each at 95°C for 15s, 52°C for 15s, and 68°C for 1min, and a final extension cycle at 68°C for 5min. Primer pairs used in the RT-PCR analysis were as: *ShGPCR1-F* and *ShGPCR1-R* for *ShGPCR1*, and *APRT2-F* and *APRT2-R* for *APRT2*. Primer sequences for the sugarcane stress-responsive marker genes *LEA*, *DHY*, *SCDR4*, *GOLS*, *ERF3*, *SOS1*, *ShNHX1*, *SsNAC23*, *CBF2*, and *ScADH3* used in the stress-tolerance assays of the *ShGPCR1*-*OE* lines are provided in Supplementary Table S1 (Nogueira et al., 2005; Iskandar et al., 2011; Reis et al., 2014; Su et al., 2014, 2020; Li et al., 2018; Devi et al., 2019; Theerawitaya et al., 2020; Brindha et al., 2021).

### Cellular Calcium Imaging and Measurements

Ca^2+^ measurements of leaf cells were performed as previously described (Li et al., 2014) with the following minor modifications: transverse sections of (5–10mm) sugarcane *ShGPCR1:OE* and NT leaves were prepared using razor blades in Tyrode solutions (145-mM NaCl, 5-mM KCl, 2-mM CaCl_2_, 1-mM MgCl_2_, 10-mM Glucose, and 20-mM HEPES, pH 7.4) with 23μM of the Ca^2+^-sensing dye Fluo-4AM (Invitrogen™ Molecular Probes™, Eugene, OR, United States) and 2.5μl of power concentrate (100x; Invitrogen™ Molecular Probes™) for 1h at room temperature. Subsequently, leaf pieces were placed on a glass coverslip and visualized under an Olympus IX71 inverted microscope attached with PTI EasyRatioPro system (HORIBA Scientific, Piscataway, NJ, United States). Changes in fluorescence of single cells were recorded with EasyRatioPro v3.4 software (HORIBA Scientific) with an excitation wavelength of 488nm and an emission wavelength of 525nm. GTP (100mM) and ionomycin (1mM; 1–3μl) were added to the leaf samples during live measurements to test their effects on Ca^2+^ release. All Ca^2+^ imaging data were analyzed with EasyRatioPro (PTI, HORIBA Scientific) software and further processed with Excel (Microsoft, Redmond, WA, United States), Igor Pro v8.0 (Wavemetrics, Lake Oswego, OR, United States) software, and graphs were plotted with Origin Pro v2020 (Originlab, Northampton, MA, United States) software. The data were presented as means±SE (*n*=number of plant cells). For all analyses, data were pooled to attain a sample size of 33–99 plant cells. Significant differences were determined using two-sample Student’s *t*-test (*p*<0.05).

## Results and Discussion

### Identification and Cloning of *ShGPCR1* From *Saccharum* spp. Hybrids

We used a combination of comparative genomics and bioinformatics tools to identify *ShGPCR1*. First, we used the DNA sequence of a *GPCR* orthologs gene from rice (LOC_Os04g51180.1) to perform a BLAST search (with cutoff *E*-value<1e-05) against the SUCEST (Vettore et al., 2003) and the National Center for Biotechnological Information (NCBI) short reads archive (Misra et al., 2007) for sugarcane sequences. We assembled a 1,407-bp sequence, hereafter called *ShGPCR1* (*Saccharum* spp. hybrids *GPCR1*), *in silico* from the retrieved hits. We compared our identified CDS to the draft monoploid sugarcane genome (Garsmeur et al., 2018; Zhang et al., 2018) that was recently released. We identified a single and perfect match (*E*-value=0) to a transcript (Sh_221E20_t000010), thus validating our targeted comparative genomics pipeline, which we applied before the release of the draft genome.

Next, we amplified the endogenous CDS from both sugarcane and energy cane using RT-PCR with primers specific to the predicted CDS. Because sugarcane and energy cane are hybrid genomes with complex ploidy, it is important to understand haplotype divergence of the homologs alleles of the corresponding gene. Hence, we isolated and sequenced several clones representing homeologs alleles of *ShGPCR1* (Table 1). We identified several alleles from the analysis of 10 independent *ShGPCR1* clones isolated from sugarcane and energy cane (Table 1; Supplementary Dataset). In general, the *ShGPCR1* alleles showed a high degree of conservation (~98% nucleotide identity) among homologs isolated from energy cane and sugarcane. These results are consistent with the high collinearity and conservation in gene structure and nucleotide sequence (~95.7% identity) among several other homologs genes observed in sugarcane, despite the complex polyploidy of the sugarcane genome (Garsmeur et al., 2011). Together, these results suggest that gene coding sequences are under purifying selection pressure in sugarcane and that homologs alleles may have all retained biologically relevant functions.

### *ShGPCR1* Is Evolutionarily Conserved With Orthologs From Rice and Maize

**Figure 1:**
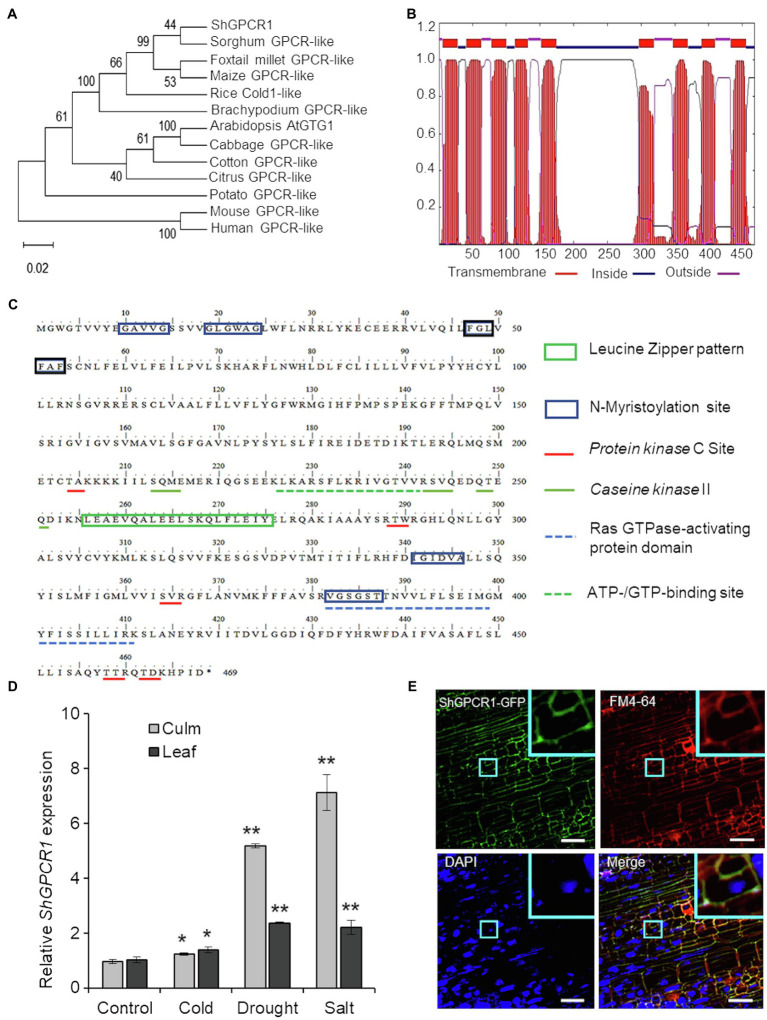
Characterization of the sugarcane *ShGPCR1*. **(A)** Phylogenetic relationship of *ShGPCR1* protein and its orthologs. Phylogenetic tree was built using the Neighbor-Joining method with pair-wise deletion of alignment gaps in MEGAX program. Values on the branches are bootstrap proportion, with the length of the branches being proportional to evolutionary distance between species. Gene abbreviations and GenBank accession numbers are as follows: *Sorghum bicolor* [sorghum; G-protein-coupled receptor (GPCR)-like protein, XP_021317902.1], *Setaria italica* (foxtail millet; GPCR-like protein, XP_012702662.1), *Zea mays* (maize; GPCR-like protein, PWZ44530.1), *Brachypodium distachyon* (GPCR-like protein, XP_003580421.1), *Oryza sativa* (COLD1, LOC_Os04g51180.1), *Arabidopsis thaliana* (GTG1, AT1G64990.1) and *Gossypium raimondii* (cotton; GPCR-like protein, Gorai.003G057600.2), *Citrus sinensis* (citrus; GPCR-like protein, XP_006494940.1), *Brassica oleracea* (cabbage; GPCR-like protein, XP_013600854.1), and *Solanum tuberosum* (potato; GPCR-like protein, XP_006357657.1). **(B)** Prediction of transmembrane regions, overall protein, and domain architecture of *ShGPCR1* using TMHMMM software. **(C)** *In silico* analysis of the sugarcane *ShGPCR1* protein. The protein motifs, patterns, and biologically significant sites in *ShGPCR1* amino acid sequence were identified using ExPaSy PROSITE database of protein domains, families, and functional sites (https://prosite.expasy.org). **(D)** Expression levels of *ShGPCR1* in sugarcane culms and leaves after cold, drought, and salinity stress treatments, as monitored by quantitative reverse transcription PCR (RT-PCR). Error bars represent the SE from three biological samples. Asterisks indicate statistically significant differences between control and treatment by Student’s *t*-test (^*^, 95% CI; *p*<0.05; and ^**^, 99% CI; *p*<0.01). **(E)** Subcellular localization of *ShGPCR1* in sugarcane. Sugarcane embryogenic leaf rolls were bombarded with *ShGPCR1::mGFP*-containing plasmid. After 10days, leaf rolls were stained with plasma membrane FM4-64 or nuclear DAPI dye and visualized using confocal microscopy. *ShGPCR1*::green fluorescent protein (mGFP) was primarily detected at the plasma membrane in the embryogenic cells (inset). Scale bar=50μm.

The predominant *ShGPCR1* allele corresponding to the 1,407-bp cDNA encoded a full-length protein with a deduced protein sequence of 468 amino acids (Supplementary Dataset), a predicted molecular mass of 53.5kDa, and an isoelectric point of 8.8. The *ShGPCR1* protein showed ~99% similarity to other GPCR-like proteins from sorghum (Sobic.006G203300.1) and maize (Zm2g129169_T01) and~96% similarity to the rice COLD1 GPCR protein (LOC_Os04g51180.1; Supplementary Figure S1). To assess the evolutionary relationship between *ShGPCR1* and other plant GPCRs, we performed a phylogenetic analysis with selected GPCRs from monocots and dicots. In general, *ShGPCR1* clustered with GPCRs from closely related monocots, such as sorghum (*Sorghum bicolor*), foxtail millet (*Setaria italica*), maize, rice, and Brachypodium (*Brachypodium distachyon*), but it was more distant from dicots, such as Arabidopsis, cabbage (*Brassica oleracea*), cotton (*Gossypium raimondii*), citrus (*Citrus sinensis*), and potato (*Solanum tuberosum*; Figure 1A).

### *ShGPCR1* Shares Characteristic Features of Plant GPCRs

One hallmark of GPCRs is their secondary structure, which consists of an N-terminal extracellular domain for membrane anchoring, 7–9 transmembrane (TM) helical-spanning domains connected by three extracellular N-terminal loops for ligand binding, three C-terminal loops in the cytosol for heterotrimeric G-protein binding, and an intracellular C-terminal tail for phosphorylation and desensitization (Wheatley et al., 2012; Ding et al., 2013). Amino acid sequence alignment of the *ShGPCR1* protein to corresponding GPCRs from monocots and dicots showed a high degree of conservation (Supplementary Figure S1). We determined the presence of the TM region by TMHMM2 prediction (Krogh et al., 2001). *ShGPCR1* possessed the nine TM helices linked by alternate intra- and extracellular loops; its N-terminal and C-terminal domains were also present on opposite sides of the membrane, as in other GPCRs (Ma et al., 2015; Figure 1B). Furthermore, we identified, by PROSITE motif analysis of *ShGPCR1*, a conserved Ras GTPase-activating protein domain and an ATP-/GTP-binding region, both important for GPCR function (Figure 1C). These domains were well characterized in the Arabidopsis GTG1, GTG2, and GCR2 proteins (Liu et al., 2007; Pandey et al., 2009; Yadav and Tuteja, 2011; Shi and Yang, 2015). The *ShGPCR1* protein also contained six *N*-myristoylation sites (Figure 1C), known to be important for co-translational or post-translational modification of GPCRs and to help anchor the protein to the membrane (De Jonge et al., 2000; Utsumi et al., 2005). These myristoylation motifs were essential for regulating signal transduction (Sessa et al., 1993; De Jonge et al., 2000) and cellular responses to high salinity (Ishitani et al., 2000). GPCRs can be phosphorylated in response to ligand stimulation by GPCR kinases and protein kinases from a diverse range of kinase families, which determines specificity in signaling outcomes (Torrecilla et al., 2007). *ShGPCR1* possessed five predicted protein kinase C and three casein kinase II phosphorylation sites (Figure 1C) that might be important for its biochemical regulation, and in relaying Ca^2+^-dependent signals (Torrecilla et al., 2007; Yadav and Tuteja, 2011). Together, these bioinformatics analyses uncover conserved and characteristic features of *ShGPCR1*.

### Steady-State Transcript Levels of *ShGPCR1* Are Upregulated by Abiotic Stresses

To investigate the *in planta* function of *ShGPCR1*, we first determined whether *ShGPCR1* transcript levels were altered in response to abiotic stress, as was reported for other plant GPCRs (Ma et al., 2015; Anunanthini et al., 2019). Quantitative RT-PCR (RT-qPCR) showed that *ShGPCR1* transcript levels were significantly induced (*p*<0.05) upon exposure to drought and salinity stress: 5.2-fold in culms and 2.4-fold in leaves (drought), and 7.1-fold in culms and 2.2-fold in leaves (salinity), relative to control tissues (Figure 1D). Cold stress modestly enhanced *ShGPCR1* expression in leaves and culms (~1.2–1.37-fold; Figure 1D) that was statistically significant (*p*<0.05) when compared to untreated tissues. *ShGPCR1* expression was also significantly induced (*p*<0.05) by exogenous ABA treatment (Supplementary Figure S2), suggesting that *ShGPCR1* responses may be ABA dependent. The increased *ShGPCR1* transcript level in response to the various stresses may lead to changes in Ca^2+^ ion flux and reactive oxygen species (ROS; Sanders et al., 1999; Munns and Tester, 2008; Mohanta et al., 2018). These often act as secondary messengers to coordinate stress-mediated signal transduction with their cognate protein kinases for adaptation to adverse conditions. In addition to the upregulation of *ShGPCR1*, the induction of GPCR proteins, such as *COLD1* from rice, maize, sorghum, and sweetcane (*Erianthus arundinaceus*) under drought, salt, or cold stress, underscores the regulatory role of GPCR proteins under abiotic stress (Ma et al., 2015; Anunanthini et al., 2019).

### *ShGPCR1* Localizes Predominantly to the Plasma Membrane

We next examined the subcellular localization of *ShGPCR1*. We cloned the full-length *ShGPCR1* CDS in frame with the CDS of green fluorescent protein (mGFP) at the *C*-terminus. We then transiently delivered the construct into sugarcane by bombardment of embryogenic leaf rolls (8days old). Confocal microscopy of bombarded leaf rolls stained with the plasma membrane-specific dye FM4-64 showed that *ShGPCR1*::mGFP co-localized with FM4-64 primarily at the plasma membrane (Figure 1E; Supplementary Figure S3). Visualization of nuclei with DAPI staining showed no overlapping nuclear localization with *ShGPCR1*::mGFP (Figure 1E; Supplementary Figure S3). The mGFP alone was detected mostly in the cytosol (Supplementary Figure S3). Together, the results suggest that *ShGPCR1* is predominantly a membrane-localized protein and supports the *in silico* predictions (Figure 1C) in a manner similar to other GPCRs (Ma et al., 2015), possibly functioning by maintaining cell membrane integrity under stress.

### Constitutive Expression of *ShGPCR1* Confers Tolerance to Multiple Abiotic Stresses

**Figure 2:**
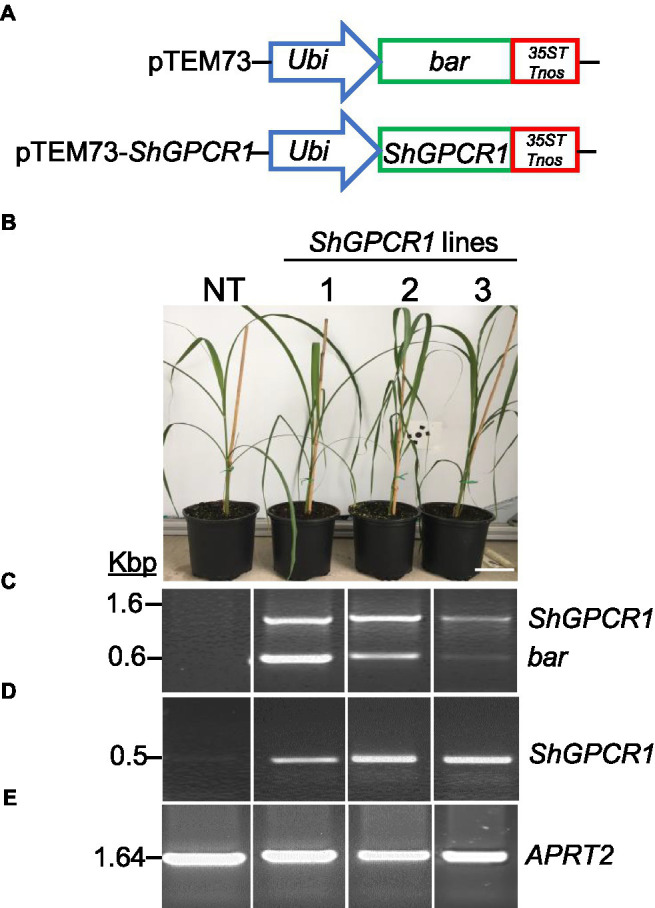
Molecular characterization of the sugarcane *ShGPCR1*-overexpressing (OE) lines. **(A)** Genetic constructs used for sugarcane transformation. **(B)** Phenotype of three independent *ShGPCR1*-*OE* lines and non-transgenic plant (NT). Scale bar=4cm. **(C)** Presence of *ShGPCR1* and *bar* (selectable marker) genes in the *ShGPCR1*-*OE* lines, as detected by PCR analysis using primers specific to the *ShGPCR1* and *bar* genes. **(D,E)** Expression analysis of *ShGPCR1* and endogenous *APRT2* genes in the *ShGPCR1*-*OE* lines, respectively, as detected by RT-PCR. *Ubi*: maize ubiquitin 1 promoter; *35ST*: terminator derived from *Cauliflower mosaic virus* 35S RNA; and *Tnos*: *Agrobacterium tumefaciens* nopaline synthase terminator. *APRT2*: sugarcane *anthranilate phosphoribosyltransferase* gene.

To further understand the *in planta* function of *ShGPCR1* in sugarcane stress signaling, we constitutively expressed a representative full-length CDS of *ShGPCR1* (EC2 allele, Table 1) under the control of the maize *Ubiquitin 1* promoter (*pUbi*) and the double terminator from *Cauliflower mosaic virus* 35S (*35ST*) and Agrobacterium (*Agrobacterium tumefaciens*) *nopaline synthase* (*Tnos*; Figure 2A), using established sugarcane DNA bombardment transformation methods (Bower et al., 1996; Ramasamy et al., 2018). We determined the presence of the *ShGPCR1* transgene and co-transformed *bar* selectable marker in transgenic sugarcane plants by PCR, with primers spanning the *pUbi*-*bar*-*35STTnos* (in pTEM73) cassette (Figure 2C; Supplementary Figure S8). We then identified several *ShGPCR1* transgenic lines with simple (1–2 insertions) or complex (>2–7 insertions) integration events, as detected by Southern blot hybridization with a full-length *ShGPCR1* CDS probe (Supplementary Figure S4). Multiple integration sites are a typical outcome of sugarcane biolistic transformation, which is largely considered preferable because of its applicability to diverse sugarcane genotypes, in contrast to *Agrobacterium*-mediated methods, which exhibit strong genotype specificity (Ramasamy et al., 2018). We confirmed overexpression of *ShGPCR1* in the transgenic plants by RT-PCR using *ShGPCR1*-specific primers. The expression of sugarcane *ANTHRANILATE PHOSPHORIBOSYLTRANSFERASE* (*APRT2*; Casu et al., 2012) was used as housekeeping gene reference (Figures 2D,E; Supplementary Figure S8). Next, we micro-propagated three independent transgenic plants (1–3) in bioreactors (Da Silva et al., 2020) that showed stable expression of *ShGPCR1* and no deleterious growth phenotypes relative to non-transgenic (NT) controls (Figure 2B) to scale up plant material for diverse stress-tolerance studies.

#### Tolerance to Drought Stress

**Figure 3:**
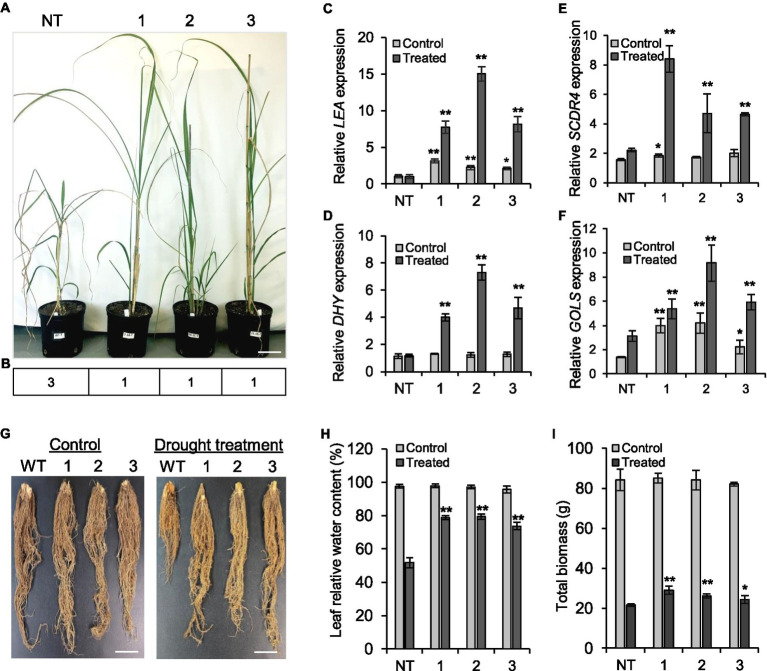
Tolerance of sugarcane *ShGPCR1*-overexpressing (OE) lines to drought stress. **(A,B)** Phenotype and severity index of three independent *ShGPCR1-OE* lines in response to drought stress, compared to non-transgenic (NT) plants. Severity index: mild=1 (few leaf curling and wilting), moderate=2 (<25–50% of leaves showing curling and wilting with concomitant necrosis), and severe=3 (>75% of leaves showing leaf curling and wilting with concomitant necrosis). **(C–F)** Expression levels of sugarcane drought stress-responsive genes, *LEA*, *DHY*, *SCDR4*, and *GOLS* in *ShGPCR1*-*OE* lines and NT plants. The relative expression of three biological replicates was normalized to the *APRT2* endogenous reference gene. **(G)** Root morphology of 4-month-old *ShGPCR1*-*OE* and NT plants after 40days of drought stress. **(H,I)** Leaf relative water content and total biomass of 4-month-old *ShGPCR1*-*OE* and NT plants after 40days of drought. Asterisks indicate statistically significant differences between NT plants and *ShGPCR1*-*OE* lines by Student’s *t*-test (^*^, 95% CI; *p*<0.05; and ^**^, 99% CI; *p*<0.01). Scale bars=4cm for **(A)** and 1cm for **(E)**.

To determine whether overexpression of *ShGPCR1* enhanced tolerance to drought stress in sugarcane, we placed 4-month-old *ShGPCR1 overexpressing (ShGPCR1-OE)* lines 1, 2, and 3 and NT plants in a temperature-controlled greenhouse and subjected them to a progressive drought treatment. This was done by withholding watering for 40days until soil moisture reached 10% and visual symptoms of wilting appeared in NT plants (Nelson et al., 2007; Ning et al., 2010). Over the course of the drought treatment, the *ShGPCR1-OE* lines showed a delay in typical stress-induced symptoms, such as leaf curling and wilting with concomitant necrosis (severity index of 1) as compared to NT plants (severity index of 3; Figures 3A,B). The *ShGPCR1:OE* lines had a well growing root system (Figure 3G) in contrast to the NT stunted root phenotype (Figure 3G) and displayed a significantly (*p*<0.05) higher dry root weight than NT plants under drought stress (Supplementary Figure S5). Furthermore, expression levels of the sugarcane drought-responsive marker genes, *LATE EMBRYOGENESIS ABUNDANT PROTEIN (LEA)*, *DEHYDRIN* (*DHY*), *DROUGHT RESPONSIVE 4 (SCDR4)*, and *GALACTINOL SYNTHASE* (*GOLS;* Reis et al., 2014), were significantly (*p*≤0.05) higher in the three *ShGPCR1-OE* lines by 7.7-, 15.1-, and 8.1-fold, 3.4-, 6.2-, and 4.0-fold, 6.2-, 2.4, and 2.4-fold, and 2.2-, 6.0-, and 2.7-fold, respectively, than in NT plants under drought stress (Figures 3C–F). The LEA proteins are important for protection of macromolecules, such as enzymes and lipids upon dehydration (Goyal et al., 2005; Reis et al., 2014), while the DHYs bind to lipid vesicles that contain acidic phospholipids capable of scavenge hydroxyl radicals (Asghar et al., 1994). The GOLS plays a key role in the accumulation of galactinol and raffinose that function as osmoprotectants in drought-stressed plants (Asghar et al., 1994; Reis et al., 2014). Our results suggest that the *ShGPCR1-OE* plants could sense the degree of water stress and activate different stress response pathways for adaptation.

Relative water content is an important parameter to determine plant drought and salinity tolerance, since water stress restricts transpiration through leaves by promoting the closure of stomata and limiting water evaporation from the leaf surface (Jin et al., 2017). We measured RWC before and after imposing water stress into *ShGPCR1-OE* lines and NT plants. Before stress, transgenic and NT plants showed no obvious differences in their RWC (Figure 3H). After 40days of water stress, the RWC of NT plants decreased to 51.80% of its pre-drought levels, whereas most transgenic lines showed a more modest decline in their RWC (78.76% for line 1, 79.23% for line 2, and 73.73% for line 3; Figure 3H). This suggests that water loss by evapotranspiration may be reduced in the *ShGPCR1-OE* lines.

Tolerance to drought is often associated with enhanced agronomic traits. At the end of the drought stress treatment, *ShGPCR1-OE* lines performed significantly (*p*<0.05) better than NT plants for several agronomic characteristics, such as total biomass (leaves and culms; Figure 3I), dry root biomass, leaf length, and culm diameter and height (Supplementary Figure S5). We observed no significant differences in growth characteristics, such as plant height, number of leaves, root length, and total biomass yield when *ShGPCR1-OE* lines and NT plants were grown under unstressed conditions (Figures 3G–I; Supplementary Figure S5). This suggests that constitutive expression of ShGPCR1 does not negatively impact growth and biomass/yield of sugarcane, while enhanced stress tolerance under water-limiting conditions.

#### Tolerance to Salinity Stress

**Figure 4:**
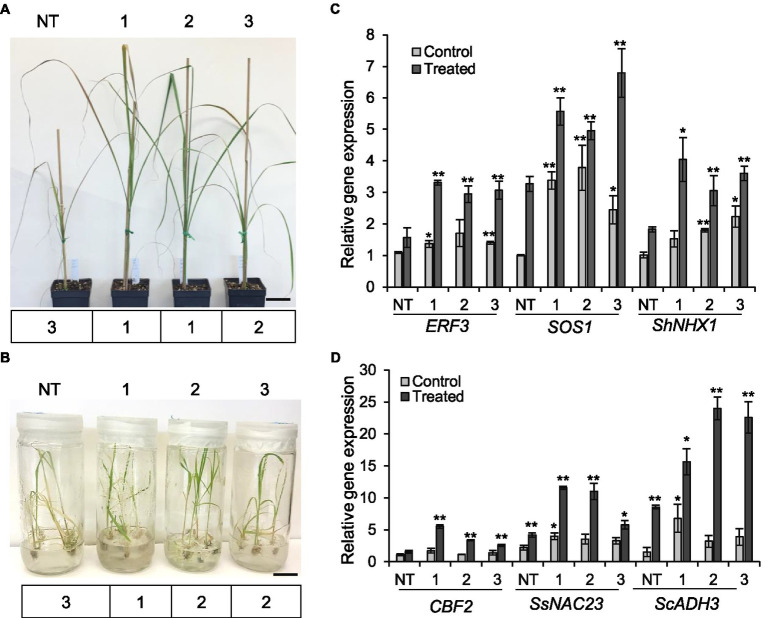
Tolerance of the sugarcane *ShGPCR1*-OE lines to salinity and cold stresses. **(A,B)** Phenotype and severity index (below the plant images) of three independent *ShGPCR1*-*OE* lines in response to salinity and cold stresses compared to non-transgenic (NT) plants, respectively. Severity index: mild=1 (few leaf curling and wilting), moderate=2 (<25–50% of leaves showing curling and wilting with concomitant necrosis), and severe=3 (>50% of leaves showing leaf curling and wilting with concomitant necrosis). Scale bars=2cm for **(A)** and 1cm for **(B)**. **(C,D)** Expression levels of sugarcane salt (*ERF3*, *SOS1*, and *ShNHX1*) and cold (*CBF2*, *SsNAC23*, and *ScADH3*) stress-responsive genes in *ShGPCR1*-*OE* lines and NT plants, respectively. The mean of *APRT2* was used as a reference to measure the relative quantification that corresponds to the mean of three biological replications. Asterisks indicate statistically significant differences between NT plants and *ShGPCR1*-*OE* lines by Student’s *t*-test (^*^, 95% CI; *p*<0.05; and ^**^, 99% CI; *p*<0.01).

We also evaluated the performance of *ShGPCR1-OE* lines and NT plants (1month old) when exposed to salinity stress in soil after irrigation with water containing 200-mM sodium chloride (200ml per 1-L pot) once a week for a period of 2weeks (Ouyang et al., 2007; Begcy et al., 2012). Under normal growth conditions, transgenic and NT plants showed no abnormal morphological phenotypes. After 14days of salinity stress, NT plants showed severe leaf chlorosis and necrosis and a collapse of aboveground tissues (severity index of 3). By contrast, *ShGPCR1-OE* lines displayed less pronounced chlorosis, necrosis, and wilting (severity index of 1–2; Figure 4A). Expression levels of the sugarcane salt-responsive marker genes, *ETHYLENE RESPONSIVE FACTOR 3* (*ERF3*; Devi et al., 2019), *SALT OVERLY SENSITIVE 1* (*SOS1*; Brindha et al., 2021), and *VACUOLAR Na+/H+ EXCHANGERS 1* (*ShNHX1*; Theerawitaya et al., 2020), were significantly higher (*p*≤0.05) in the three *ShGPCR1*-*OE* lines by 2.1-, 1.9-, and 2.0-fold, 2.3-, 1.7-, and 3.5-fold, and 2.2-, 1.2-, and 1.7-fold, respectively, than in NT plants under salinity stress (Figure 4C). In response to salinity stress, low salt levels are maintained in the cytoplasm by removal of sodium (Na^2+^) through transporters from the cytoplasm into the vacuole or out of the cell; this transport is catalyzed by Na^+^/H^+^ exchangers (antiporters), such as SOS1 and NHX1 (Shi et al., 2003; Qiu et al., 2004). Our results suggest that the adaptation of the *ShGPCR1-OE* plants to high salinity could be mediated through the activation of SOS1 and NHX1 antiporters.

#### Tolerance to Cold Stress

We next evaluated the performance of *ShGPCR1-OE* and NT plants (1month old) when exposed to 4°C for 3weeks, followed by −5°C for 4h. Cold tolerance assays were performed *in vitro* (seedlings grown in tissue culture on nutrient medium) and in soil (potted plants) in environment-controlled growth chambers (Rivero et al., 2001; Belintani et al., 2012; Park et al., 2015). *ShGPCR1-OE* lines showed a higher survival rate *in vitro* and *in vivo* (severity index of 12; Figure 4B; Supplementary Figures S6A,B) compared to NT plants, which exhibited severe wilting and yellowing of leaves (severity index of 3) upon cold stress (Figure 4B; Supplementary Figures S6A,B). Expression of the sugarcane cold-responsive marker genes, *NAM*,/*ATAF1/2*,/*CUC2* (*SsNAC23*; Nogueira et al., 2005), *COLD BINDING FACTOR 2* (*CBF2*; Mirkov et al., 2013), and *ALCOHOL DEHYDROGENASE 3* (*ScADH3*; Su et al., 2020), was significantly (*p*≤0.05) higher in the *ShGPCR1-OE* lines by 2.8-, 2.7-, and 1.4-fold, 3.6-, 2.2-, and 1.7-fold, and 7.1-, 15.4-, and 14.0-fold, respectively, than in NT plants under cold stress (Figure 4D).

Abiotic stress induces the production of ROS, which causes a redox imbalance and oxidative damage to cell structure and functioning. The antioxidant enzymes, catalase (CAT), superoxide dismutase (SOD), and peroxidase (POX) scavenge the excess amounts of ROS produced in the cell during abiotic stress (Su et al., 2014; Sofo et al., 2015). In this study, we show that the expression level of the *CAT* gene (Su et al., 2014) in the *ShGPCR1*-OE lines significantly (*p*<0.05) increased upon drought (0.9-, 1.1-, and 2.1-fold), salinity (2.2-, 3.0-, and 3.1-fold), and cold (0.5-, 2.4-, and 3.5-fold), compared to NT plants (Supplementary Figure S7), suggesting that overexpression of *ShGPCR1* enhances the ROS-scavenging capacity, thereby decreasing ROS damage under stress conditions.

In summary, our findings from stress-tolerance assays indicate that overexpression of *ShGPCR1* in sugarcane conferred tolerance to drought, salinity, and cold stresses without negatively affecting plant growth, as it was also shown for chilling tolerant *COLD1*-overexpressing rice (Ma et al., 2015) and salt tolerant Arabidopsis overexpressing cotton *TOM1* (Lu et al., 2018). Further, the stress-tolerance phenotype of *ShGPCR1-OE* lines corresponded with the induction of sugarcane drought, salinity, and cold stress-responsive marker genes, such as *LEA*, *DHY*, *SCDR4*, and *GOLS* (drought), *ERF3*, *SOS1*, and *ShNHX1* (salinity), and *SsNAC23*, *CBF2*, and *ScADH3* (cold; Figures 3, 4).

### *ShGPCR1* Enhances Cellular Calcium Levels in Response to GTP

**Figure 5:**
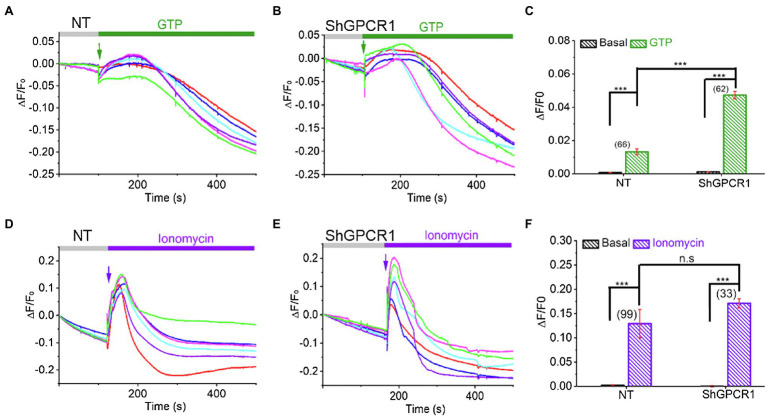
*ShGPCR1*-mediated Ca^2+^ release in sugarcane leaf cells. Representative Ca^2+^ imaging trace showing guanidine triphosphate (GTP)-induced Ca^2+^ release in *ShGPCR1;OE* **(A)** and NT **(B)** sugarcane leaf cells. **(C)** Bar graph analysis of data shown in **(A,B)** depicts maximum Ca^2+^ release after GTP application. Representative Ca^2+^ imaging trace showing ionomycin induced global Ca^2+^ release in *ShGPCR1:OE* **(D)** and NT **(E)** sugarcane leaf cells. **(F)** Bar graph analysis of data shown in **(D,E)** depicts maximum Ca^2+^ release after ionomycin application. Different color traces in the graphs **(A,B,D,E)** reflect the GTP-induced Ca^2+^ responses of multiple independent cells in a given measurement. Statistical analysis was performed using Student’s *t*-test. The triple asterisk (^***^) represents statistical significance of differences between treated and control at 99.9% CI (*p*<0.0001; n.s., not significant). The number of cells (N) from 1 to 3 independent measurements is provided in parentheses in **(C,F)**.

**Figure 6:**
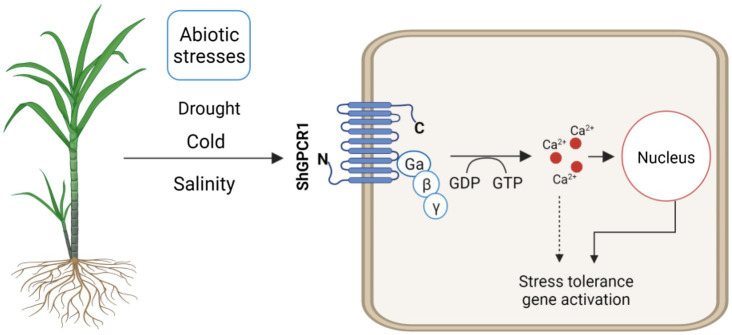
Hypothetical model of *ShGPCR1* function in sugarcane to confer tolerance to multiple abiotic stresses. *ShGPCR1* overexpression leads to accumulation in steady-state transcript levels of multiple drought, cold, and salinity stress-associated genes, both basal and under stressed conditions. The transmembrane-localized *ShGPCR1* activates a GTP-dependent Ca^2+^ increase in sugarcane cells, likely contributing to the stress-tolerance responses.

Ca^2+^ is a critical divalent cation for plant cells. The intracellular Ca^2+^ level is very low in cytosol (~100–200nM), while a high level of Ca^2+^ can be found in apoplasts (10μM–10mM), the vacuole (0.2mM to 1–5mM), and the endoplasmic reticulum (varies between 50and 500μM; Stael et al., 2012). Growing evidence indicates that changes in intracellular Ca^2+^ level or sensitivity play crucial roles in plants’ biotic and abiotic stress responses (Huda et al., 2013). Specifically, GPCRs have been known to trigger Ca^2+^ influx and signaling in plants (Ma et al., 2015). To test whether *ShGPCR1* affects cellular Ca^2+^ fluxes, we measured Ca^2+^ levels in an *ShGPCR1-OE* sugarcane line using the potent Ca^2+^-sensitive fluorescent dye Fluo-4AM (Ma et al., 2015). We found a significant enhancement in the Ca^2+^ release in response to GTP between *ShGPCR1:OE* and NT leaf cells (*ShGPCR1:OE*: 0.048±0.002; NT: 0.013±0.002; *p*=2.5E^−22^; Figures 5A–C). To further investigate whether there is any difference in the global Ca^2+^ levels between *ShGPCR1:OE* and NT leaf cells, we performed Ca^2+^ imaging in the presence of ionomycin, a bonafide calcium ionophore known to increase global Ca^2+^ levels in cells (Qiu et al., 2020). Our results showed that there was no significant difference in the overall Ca^2+^ level between *ShGPCR1:OE* and NT leaf cells (*ShGPCR1:OE*: 0.17±0.029; NT: 0.13±0.009; *p*=0.15; Figures 5D–F). Taken together, these results indicate that *ShGPCR1* affects intracellular Ca^2+^ levels in response to GTP. Given that *ShGPCR1* predominantly localized to the plasma membrane, the *ShGPCR1*-mediated Ca^2+^ increase in response to GTP could be due to an influx from an apoplast source to the cytosol. This increase in intracellular Ca^2+^ levels *via ShGPCR1* could further trigger a signaling cascade to impart abiotic stress tolerance (Figure 6).

## Conclusion

Membrane-bound receptor proteins, such as GPCRs, are associated with signal perception and transduction and control plant growth, development, and response to stresses. In this study, we identified and characterized the functions of *ShGPCR1* in abiotic stress tolerance in sugarcane, a major sugar, and bioenergy feedstock. The upregulation of *ShGPCR1* expression by drought, salinity, and cold and the enhanced tolerance of *ShGPCR1-OE* lines to the respective stresses show that *ShGPCR1* is a central player in mediating responses to diverse environmental stressors in sugarcane. The respective sugarcane transgenic lines may be further leveraged to enhance sugarcane production in marginal environments with fewer resources.

## Data Availability Statement

The datasets presented in this study can be found in online repositories. The names of the repository/repositories and accession number(s) can be found in the article/Supplementary Material.

## Author Contributions

MR, MD, CV-B, JD, NS, and KM designed the experiments. MR, MD, CV-B, VM, JL, CP, and TS conducted the experiments. MR, MD, CV-B, VM, JL, SI, NS, and KM analyzed the data and prepared the manuscript. NS, JD, and KM supervised the study. All authors contributed to the article and approved the submitted version.

## Funding

This research was supported in part by funds from the USDA-NIFA (2016-67013-24738 and HATCH 1023984) to KM; Texas A&M AgriLife Research Seed Grants (124738-96210 and 124190-96210) to KM and JD, and College of Sciences, University of Texas-Rio Grande Valley (RGV) startup fund; and University of Texas System Rising STARs Award to NS.

## Conflict of Interest

The authors declare that the research was conducted in the absence of any commercial or financial relationships that could be construed as a potential conflict of interest.

## Publisher’s Note

All claims expressed in this article are solely those of the authors and do not necessarily represent those of their affiliated organizations, or those of the publisher, the editors and the reviewers. Any product that may be evaluated in this article, or claim that may be made by its manufacturer, is not guaranteed or endorsed by the publisher.

## Footnotes

^1^ https://web.expasy.org/translate/

^2^ www.ncbi.nlm.nih.gov/BLAST

^3^ www.ebi.ac.uk/clustalw

^4^ http://bioinfo.ut.ee/primer3-0.4.0/primer3/
