# Supplementary material for: A Sugarcane G-Protein-Coupled Receptor, ShGPCR1, Confers Tolerance to Multiple Abiotic Stresses
Source: Front Plant Sci. 2021 Nov 11;12:745891. doi: 10.3389/fpls.2021.745891 (PMC8919185; doi:10.3389/fpls.2021.745891)
Supplement: Supplementary file 1 [file Data_Sheet_1.pdf]

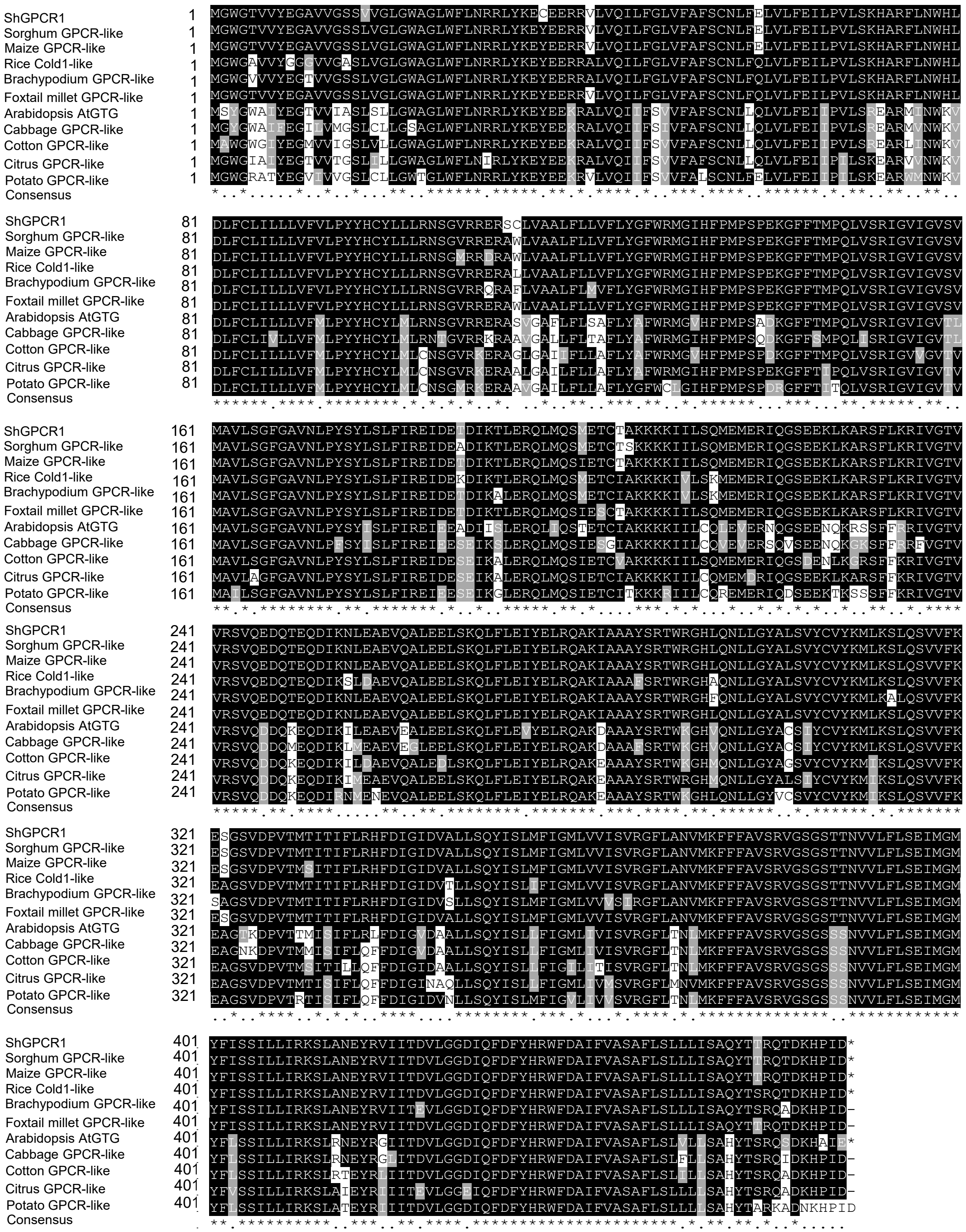

Supplemental Figure S1. Sequence homology of the sugarcane *ShGPCR1* protein with its orthologs in plants. Amino acid sequences were aligned using the BOXSHADE multiple sequence alignment program (version 3.21). Gene abbreviations and GenBank accession numbers are as follows: *Sorghum bicolor* (sorghum) (GPCR-like protein, XP_021317902.1), *Oryza sativa* (COLD1, LOC_Os04g51180.1), *Brachypodium distachyon* (GPCR-like protein, XP_003580421.1), *Zea mays* (maize) (GPCR-like protein, PWZ44530.1), *Setaria italica* (foxtail millet) (GPCR-like protein, XP_012702662.1), *Arabidopsis thaliana* (GTG1, AT1G64990.1), *Brassica oleracea* (cabbage) (GPCR-like protein, XP_013600854.1), *Gossypium raimondii* (cotton) (GPCR-like protein, Gorai.003G057600.2), *Citrus sinensis* (citrus) (GPCR-like protein, XP_006494940.1) and *Solanum tuberosum* (potato) (GPCR-like protein, XP_006357657.1). The consensus (cons.) symbols are indicated below the alignments with 100% identical residues indicated by black shading and an asterisk (*). Amino acids with ≥50% identity is shaded in gray and marked with a period.

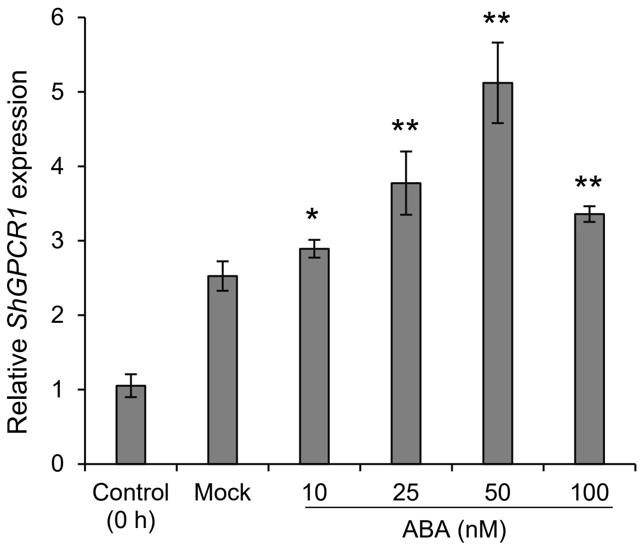

Supplemental Figure S2. Expression levels of *ShGPCR1* in detached sugarcane leaves after ABA treatment (10 h), as monitored by quantitative RT-PCR. Error bars represent the SE from three biological samples. Asterisks indicate statistically significant differences between control and treatment by Student's t-test (*, 95% confidence interval; $p < 0.05$; and **, 99% confidence interval; $p < 0.01$).

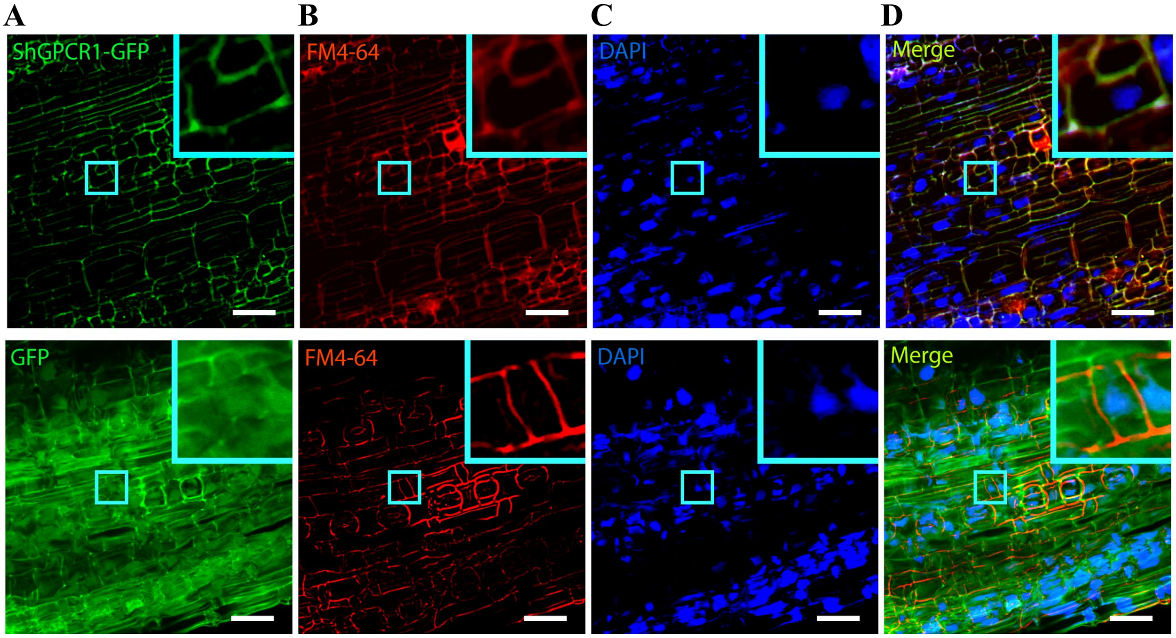

Supplemental Figure S3. Subcellular localization of *ShGPCR1* in sugarcane. Sugarcane embryogenic leaf rolls were bombarded either with *ShGPCR1::GFP* or *GFP* containing plasmids. (**A-D**) Visualization of *ShGPCR1*::GFP (top panels) or GFP (bottom panel) localization. *ShGPCR1*::GFP was predominantly localized to the plasma membrane (top inset in **A**), whereas GFP alone was detected predominantly in the cytosol (bottom inset in **A**). (**B-C**) The cells were stained with DAPI and FM4-64 to mark membrane and nuclear structures, respectively. (**D**) A merged image of all channels: GFP, FM4-64 and DAPI. Scale bar = 50 μm.

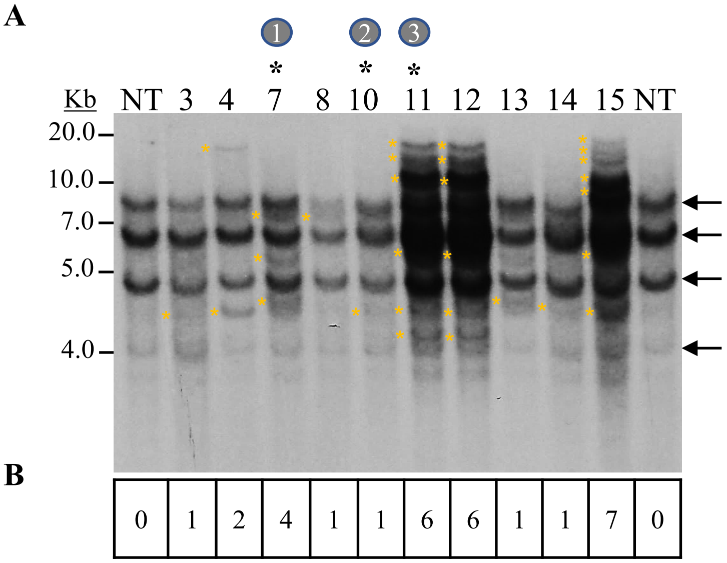

Supplemental Figure S4. Stable integration of *ShGPCR1* in the *ShGPCR1*-overexpressing (OE) lines as determined by Southern blot analysis. (**A**) *ShGPCR1* integration pattern of 10 lines. (**B**) Number of possible integrations (as indicated by yellow asterisks) of *ShGPCR1* in the sugarcane genome. The full-length *ShGPCR1* was used as a probe for hybridization. The signals observed in both non-transgenic (NT) plants and transgenic lines in the Southern blot are a result of the sugarcane endogenous *ShGPCR* hybridizing to the *ShGPCR1* probe. The hybridization bands found in NT are indicated by a black arrow. Black asterisks (*) and the number (7, 10, 11) indicate the selected *ShGPCR1-OE* lines used for characterization in abiotic stress tolerance assays.

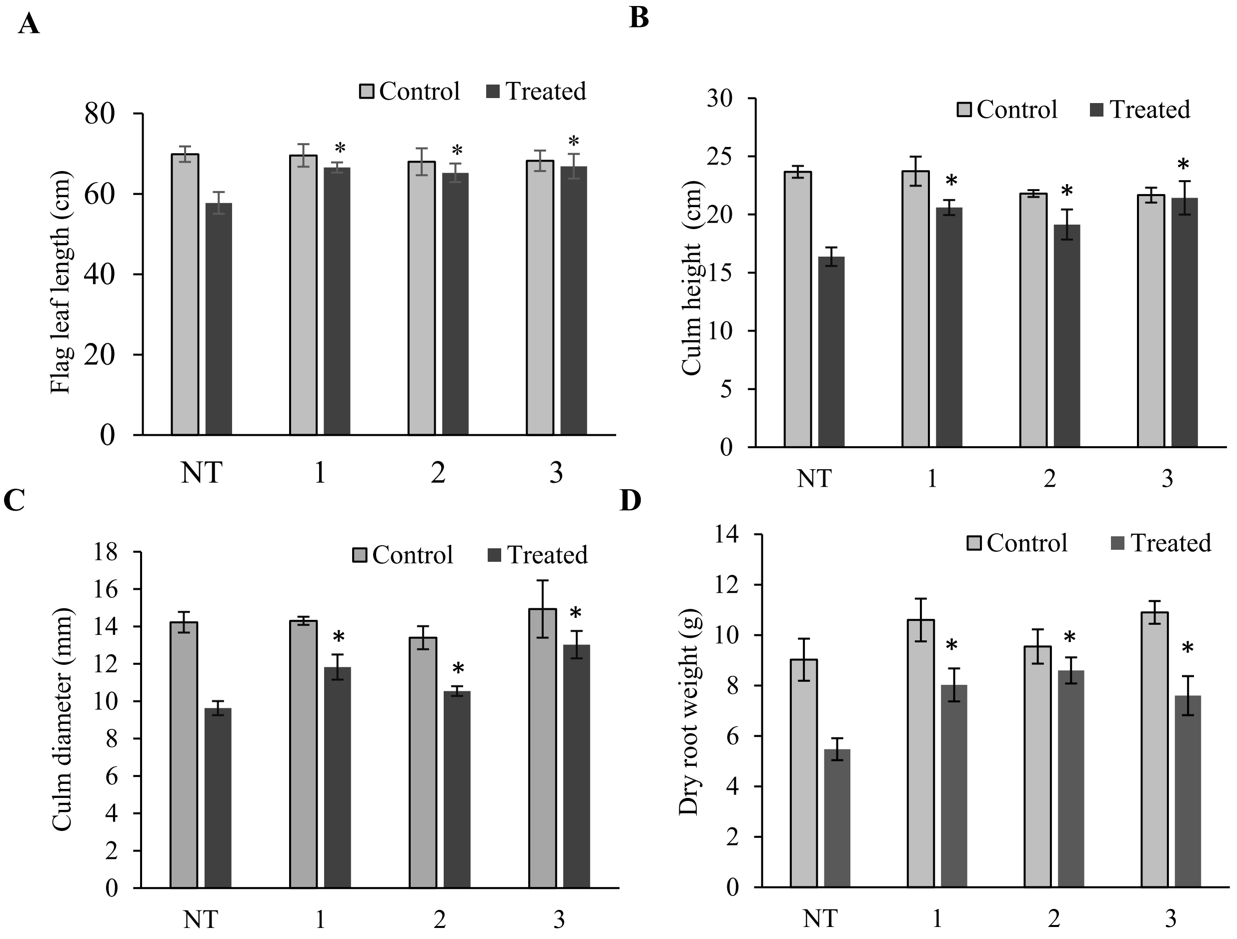

Supplemental Figure S5. Assessment of agronomic characters of three independent sugarcane *ShGPCR1*-overexpressing lines (1, 2 and 3) and non-transgenic (NT) plants (4-month-old) after 40 d of drought. (**A**) Flag leaf length, (**B**) culm height, (**C**) culm diameter, and (**D**) dry root weight. Asterisks indicate statistically significant differences between NT plants and *ShGPCR1-OE* lines by Student's t-test (*, 95% confidence interval; $p < 0.05$).

A

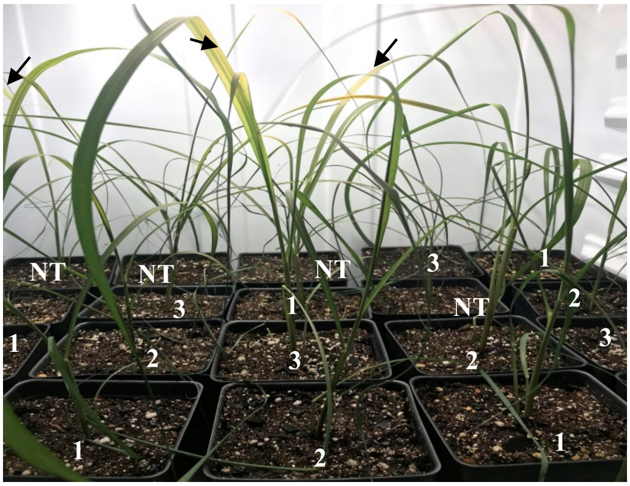

B

Severity index	4	1	2	2

Supplemental Figure S6. Tolerance of the sugarcane *ShGPCR1*-overexpressing (OE) lines to cold stress. (**A, B**) Phenotype and severity index of independent *ShGPCR1-OE*o lines (1, 2 and 3) in response to cold stress, compared to non-transgenic (NT) plants. For chilling stress tolerance assays, two-month-old greenhouse-grown *ShGPCR1:OE* and NT plants were treated at 4° C for three weeks followed by –5° C for 4 h in a temperature-controlled growth chamber. Scale bar = 2.5 cm.

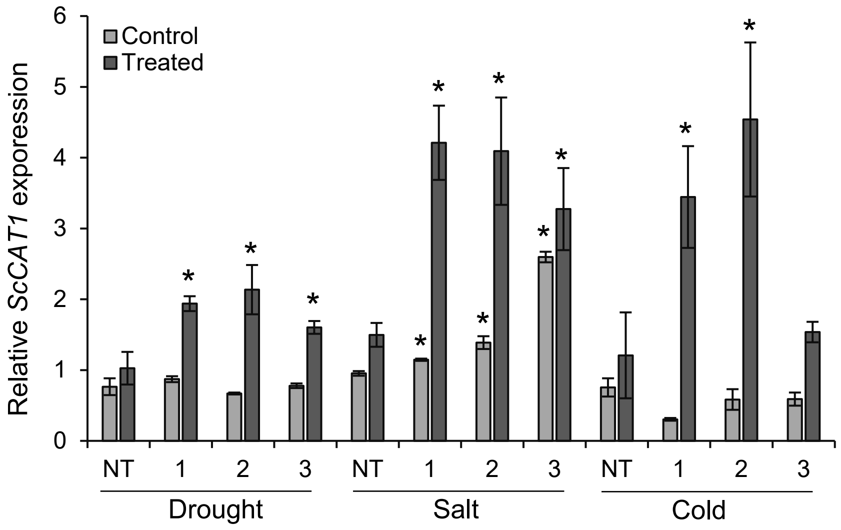

Supplemental Figure S7. Expression levels of *Saccharum catalase1* (*ScCAT1*) gene in *ShGPCR1*-overexpression lines and NT plants after exposure to drought, salt, and cold treatment, as monitored by quantitative RT-PCR. Error bars represent the SE from three biological samples. Asterisks indicate statistically significant differences between control and treatment by Student's t-test (*, 95% confidence interval; p < 0.05).

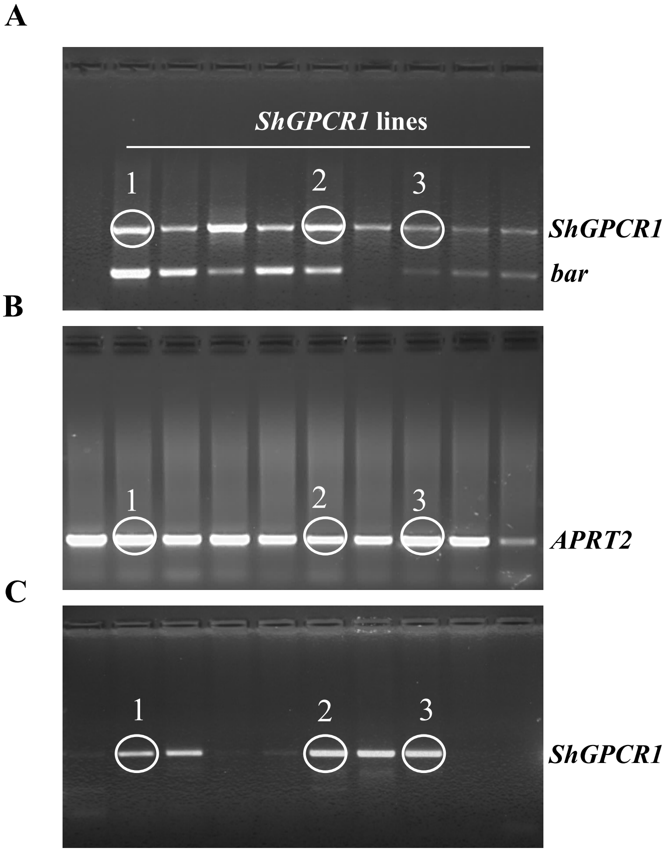

Supplemental Figure S8. Uncropped raw agarose gel images used to prepare **Figures 2C**, **D** and **E.** Numbers correspond to the numbers of *ShGPCR1*-overexpressing lines (1, 2 and 3) selected for characterization in abiotic stress tolerance assays.

Supplementary Tables

A *Saccharum spp.* G-protein-coupled receptor, ShGPCR1, confers tolerance to multiple abiotic stresses.

Short Title: ShGPCR1 confers tolerance to multiple abiotic stresses in sugarcane.

Manikandan Ramasamy[1], Mona B. Damaj[1], Carol Vargas-Bautista[1#], Victoria Mora[1], Jiaxing Liu[1], Carmen S. Padilla[1], Sonia Irigoyen[1], Tripti Sani[2], Nirakar Sahoo[2], Jorge A. DaSilva[1,3], and Kranthi K. Mandadi[1,4*]

[1]Texas A&M AgriLife Research and Extension Center, 2415 East US Highway 83, Weslaco, Texas 78596

[2]Department of Biology, University of Texas Rio Grande Valley, Edinburg, Texas 78539

[3]Department of Soil & Crop Sciences, Texas A&M University, 2474 TAMU, College Station, Texas 77843

[4]Department of Plant Pathology & Microbiology, Texas A&M University, College Station, Texas 77843

[#]Present affiliation: College of Medicine, 8447 Riverside Parkway, Texas A&M University, Bryan, Texas 77807

* The author responsible for contact and correspondence: kkmandadi@tamu.edu

Supplementary Table S1. Primers used for quantifying expression levels of *ShGPCR1* and sugarcane stress-responsive marker genes. *LEA*: *LATE EMBRYOGENESIS ABUNDANT PROTEIN*; *DHY*: *DEHYDRIN*; *SCDR4*: *Saccharum DROUGHT RESPONSIVE 4*; *GOLS*: *GALACTINOL SYNTHASE*; *ERF3*: *ETHYLENE RESPONSIVE FACTOR 3*; *SOS1*: *SALT OVERLY SENSITIVE 1*; ShNHX1: *Saccharum VACUOLAR NA+/H+ ANTIPORTER 1*, *SsNAC23*: *NAM/ATAF1/2/CUC2*; *CBF2*: *COLD BINDING FACTOR 2*; *ScADH3*: *Saccharum ALCOHOL DEHYDROGENASE 3*; and *ScCAT1*: *Saccharum CATALASE 1*.

Primer	Sequence (5' to 3')	Reference
**ShGPCR1-CF**	CGGGATCCATGGGGTGGGGCACAGTGGTT	Present study
**ShGPCR1-CR**	GCCACGTGTCAATCAATCGGATGCTTGTCT	Present study
LEA-F	TGTTTCTCCATCTCCCGAGTG	(Reis et al., 2014)
LEA-R	CATGGCAGGGTCTCTCAAGC	(Reis et al., 2014)
DHY-F	ACCAGTACGGCAATCCAGTTG	(Reis et al., 2014)
DHY-R	CGGAGCGATGCAGGATG	(Reis et al., 2014)
**SCDR4-F**	ACGAGGAGCAGAGCTATGGT	(Reis et al., 2014)
**SCDR4-R**	CGGTTTTGGCTTCGGGTAA	(Reis et al., 2014)
**GOLS-F**	AGTACAGGCCGATCCCGAAC	(Iskandar et al., 2011)
**GOLS-R**	GCAGTAGTGCACGGCCTTC	(Iskandar et al., 2011)
ERF3-F	GATGGTCATGTGATTGCCGC	(Devi et al., 2019)
ERF3-R	CAAAGGCGCAAATCTGGCAG	(Devi et al., 2019)
**SOS1-F**	GAGGGTTCTCATAGCTGAAAGG	(Brindha et al., 2021)
**SOS1-R**	GCGTGATTAGAGTCAGGTTCTC	(Brindha et al., 2021)
**ShNHX1-F**	TGGTGGGCTGGACTGATGAGAGGCG	(Theerawitaya et al., 2020)
**ShNHX1-R**	TGCCGTGCAGCTGAGTGTGTCCAGA	(Theerawitaya et al., 2020)
SsNAC23-F	CGAGAAGACCAACTGGATCA	(Nogueira et al., 2005)
SsNAC23-R	GCCCTCCCTTCTTGTTGTAG	(Nogueira et al., 2005)
CBF2-F	AGCGATGTATGGAGACTTGGC	(Mirkov et al., 2013)
CBF2-R	CTTGTGTGAGGTGGATGCGAT	(Mirkov et al., 2013)
**ScADH3-F**	AACCTTCTTCGGCAACTA	(Su et al., 2020)
**ScADH3-R**	CTCCAGCTCCTTCTTCAT	(Su et al., 2020)
**ScCAT1-F**	CTCTGCTCCTCCAATCCC	(Su et al., 2014)
**ScCAT1-R**	GAGTGACCTCAAAGAAACCCT	(Su et al., 2014)

References

Brindha, C., Vasantha, S., Raja, A.K., and Tayade, A.S. (2021). Characterization of the Salt Overly Sensitive pathway genes in sugarcane under salinity stress. *Physiol. Plant.* 171**,** 677-687.

Devi, K., Prathima, P., Gomathi, R., Manimekalai, R., Lakshmi, K., and Selvi, A. (2019). Gene expression profiling in sugarcane genotypes during drought stress and rehydration. *Sugar Tech* 21**,** 717-733.

Iskandar, H.M., Casu, R.E., Fletcher, A.T., Schmidt, S., Xu, J., Maclean, D.J., Manners, J.M., and Bonnett, G.D. (2011). Identification of drought-response genes and a study of their expression during sucrose accumulation and water deficit in sugarcane culms. *BMC Plant Biol.* 11**,** 1-14.

Mirkov, T.E., Beyene, G., and Damaj, M. (2013). "Materials, systems, organisms, and methods for enhancing abiotic stress tolerance, increasing biomass, and/or altering lignin composition". Google Patents).

Nogueira, F.T.S., Schlögl, P.S., Camargo, S.R., Fernandez, J.H., De Rosa, V.E., Pompermayer, P., and Arruda, P. (2005). SsNAC23, a member of the NAC domain protein family, is associated with cold, herbivory and water stress in sugarcane. *Plant Sci.* 169**,** 93-106.

Reis, R.R., Da Cunha, B.A., Martins, P.K., Martins, M.T., Alekcevetch, J.C., Chalfun, A., Jr., Andrade, A.C., Ribeiro, A.P., Qin, F., Mizoi, J., Yamaguchi-Shinozaki, K., Nakashima, K., Carvalho Jde, F., De Sousa, C.A., Nepomuceno, A.L., Kobayashi, A.K., and Molinari, H.B. (2014). Induced over-expression of AtDREB2A CA improves drought tolerance in sugarcane. *Plant Sci.* 221**,** 59-68.

Su, W., Ren, Y., Wang, D., Su, Y., Feng, J., Zhang, C., Tang, H., Xu, L., Muhammad, K., and Que, Y. (2020). The alcohol dehydrogenase gene family in sugarcane and its involvement in cold stress regulation. *BMC Genomics* 21**,** 1-17.

Su, Y., Guo, J., Ling, H., Chen, S., Wang, S., Xu, L., Allan, A.C., and Que, Y. (2014). Isolation of a novel peroxisomal catalase gene from sugarcane, which is responsive to biotic and abiotic stresses. *PLoS One* 9**,** e84426.

Theerawitaya, C., Tisarum, R., Samphumphuang, T., Singh, H.P., Takabe, T., and Cha-Um, S. (2020). Expression levels of vacuolar ion homeostasis-related genes, Na+ enrichment, and their physiological responses to salt stress in sugarcane genotypes. *Protoplasma* 257**,** 525-536.

Supplementary Dataset

Short Title: ShGPCR1 confers tolerance to multiple abiotic stresses in sugarcane.

Manikandan Ramasamy[1], Mona B. Damaj[1], Carol Vargas-Bautista[1#], Victoria Mora[1], Jiaxing Liu[1], Carmen S. Padilla[1], Sonia Irigoyen[1], Tripti Sani[2], Nirakar Sahoo[2], Jorge A. DaSilva[1,3], and Kranthi K. Mandadi[1,4*]

[1]Texas A&M AgriLife Research and Extension Center, 2415 East US Highway 83, Weslaco, Texas 78596

[2]Department of Biology, University of Texas Rio Grande Valley, Edinburg, Texas 78539

[3]Department of Soil & Crop Sciences, Texas A&M University, 2474 TAMU, College Station, Texas 77843

[4]Department of Plant Pathology & Microbiology, Texas A&M University, College Station, Texas 77843

[#]Present affiliation: College of Medicine, 8447 Riverside Parkway, Texas A&M University, Bryan, Texas 77807

* The author responsible for contact and correspondence: kkmandadi@tamu.edu

>SC1-Coding Sequences (CDS)
ATGGGGTGGGGCACAGTGGTTTACGAGGGCGCGGTCGTCGGCTCGTCGCTGGTGGGGCTGGGCTGGGCGGGGCTGTGGTTCCTGA
ACCGGCGGCTGTACAAGGAGTACGAGGAGCGGCGGGTGCTGGTGCAGATCCTCTTCGGCCTCGTCTTCGCCTTCTCCTGCAACCTC
TTCGAGCTCGTTCTCTTCGAGATCCTCCCCGTCCTCTCCAAGCATGCGCGCTTCCTCAACTGGCACCTCGACCTCTTCTGCCTCATC
CTCCTCCTCGTCTTCGTGCTCCCCTACTACCACTGCTATCTTCTGCTCCGTAACTCAGGGGTGAGGAGGGAGCGGTCGTGCCTCGTC
GCGGCGCTCTTTCTGCTGGTCTTCCTATACGGGTTCTGGCGCATGGGGATTCACTTCCCCATGCCTTCACCAGAGAAGGGTTTTTT
ACGATGCCGCAGCTGGTCAGTAGGATTGGGGTGATTGGAGTGAGTGTCATGGCTGTTCTTTCTGGTTTTGGTGCTGTCAATCTGCC
ATACAGTTATCTGTCACTCTTCATCAGGGAAATTGATGAAACAGACATCAAAACCTTGGAACGGCAGCTGATGCAATCCATGGAG
ACATGTACTGCTAAGAAGAAGAAAATTATTTTGTCCCAGATGGAGATGGAGAGGATTCAAGGATCAGAGGAGAAGCTAAAGGCC
AGATCGTTTCTGAAGCGTATAGTGGGAACTGTTGTCAGATCTGTGCAGGAAGATCAAACTGAGCAGGATATAAAAAACTTAGAAG
CAGAAGTCCAGGCACTGGAAGAGCTTTCCAAACAGCTGTTCCTTGAGATATATGAACTCCGTCAGGCTAAGATAGCTGCTTCGTA
TTCTCGAACGTGGAGAGGGCATCTTCAGAATCTACTTGGATATGCTTTGTCGGTGTATTGTGTTTATAAGATGCTCAAGTCCTTGC
AGAGTGTAGTCTTTAAGGAGTCAGGCTCTGTTGATCCTGTAACAATGACAATAACGATTTTCCTGAGACATTTTGACATTGGCATT
GATGTTGCACTGTTATCTCAGTATATATCTTTGATGTTCATTGGGATGTTGGTTGTCATATCTGTTCGAGGTTTCTTGGCTAATGTTA
TGAAGTTCTTCTTCGCCGTTTCTAGAGTTGGGAGTGGGTCAACAACTAATGTTGTCCTTTTCCTATCAGAGATCATGGGCATGTACT
TCATATCTTCCATTCTTCTTATAAGAAAAAGCCTGGCAAATGAATATAGGGTGATCATTACTGATGTTTTGGGTGGTGATATCCAA
TTTGACTTCTACCACCGCTGGTTTGATGCTATATTTGTGGCTAGTGCGTTCCTGTCCTTGCTTCTGATTTCTGCCCAATACACCACCA
GGCAAACAGACAAGCATCCGATTGATTGA

>SC1-Amino Acid Sequences
MGWGTVVYEGAVVGSSLVGLGWAGLWFLNRRLYKEYEERRVLVQILFGLVFAFSCNLFELVLFEILPVLSKHARFLNWHLDLFCLILL
LVFVLPYYHCYLLLRNSGVRRERSCLVAALFLLVFLYGFWRMGIHFPMPSPEKGFFTMPQLVSRIGVIGVSVMAVLSGFGAVNLPYSYL
SLFIREIDETDIKTLERQLMQSMETCTAKKKKIILSQMEMERIQGSEEKLKARSFLKRIVGTVVRSVQEDQTEQDIKNLEAEVQALEELSK
QLFLEIYELRQAKIAASYSRTWRGHLQNLLGYALSVYCVYKMLKSLQSVVFKESGSVDPVTMTITIFLRHFDIGIDVALLSQYISLMFIG
MLVVISVRGFLANVMKFFFAVSRVGSGSTTNVVLFLSEIMGMYFISSILLIRKSLANEYRVIITDVLGGDIQFDFYHRWFDAIFVASAFLSL
LLISAQYTTRQTDKHPID*

>SC2- CDS
ATGGGGTGGGGCACAGTGGTTTACGAGGGCGCGGTCGTCGGCTCGTCCCTGGTGGGGCTGGGCTGGGCGGGGCTGTGGTTCCTGA
ACCGGCGGCTGTACAAGGAGTACGAGGAGCGGCGGGTGCTGGTGCAGATCCTCTTCGGCCTCGTCTTCGCCTTCTCCTGCAACCTC
TTCGAGCTCGTTCTCTTCGAGATCCTCCCCGTCCTCTCCAAGCATGCGCGCTTCCTCAACTGGCACCTCGACCTCTTCTGCCTCATC
CTCCTCCTCGTCTTCGTGCTCCCCTACTACCACTGCTATCTTCTGCTCCGTAACTCAGGGGTGAGGAGGGAGCGGTCGTGCCTCGTC
GCGGCGCTCTTTCTGCTGGTCTTCCTATACGGGTTCTGGCGCATGGGGATTCACTTCCCCATGCCTTCACCAGAGAAGGGTTTTTT
ACGATGCCGCAGTTGGTCAGTAGGATTGGGGTGATTGGAGTGAGTGTCATGGCTGTTCTTTCTGGTTTTGGTGCTGTCAATCTGCC
ATACAGTTATCTGTCACTCTTCATCAGGGAAATTGATGAAACAGACATCAAAACCTTGGAACGGCAGCTGATGCAATCCATGGAG
ACATGTACTGCTAAGAAGAAGAAAATTATTTTGTCCCAGATGGAGATGGAGAGGATTCAAGGATCAGAGGAGAAGCTAAAGGCC
AGATCGTTTCTGAAGCGTATAGTGGGAACTGTTGTCAGATCTGTGCAGGAAGATCAAACTGAGCAGGATATAAAAAACTTAGAAG
CAGAAGTCCAGGCACTGGAAGAGCTTTCCAAACAGCTGTTCCTTGAGATATATGAACTACGTCAGGCTAAGATAGCTGCTGCGTA
TTCTCGAACGTGGAGAGGGCATCTTCAGAATCTACTTGGATATGCTTTGTCGGTGTATTGTGTTTATAAGATGCTCAAGTCCTTGC
AGAGTGTAGTCTTTAAGGAGTCAGGCTCTGTTGATCCTGTAACAATGACAATAACGATTTTCCTGAGACATTTTGACATTGGCATT
GATGCTGCACTGTTATCTCAGTATATATCTTTGATGTTCATTGGGATGTTGGTTGTCATATCTGTTCGAGGTTTCTTGGCTAATGTT
ATGAAGTTCTTCTTCGCTGTTTCTAGAGTTGGGAGTGGGTCGACAACTAATGTTGTCCTTTTCCTATCAGAGATCATGGGCATGTAC
TTCATATCTTCCATTCTTCTTATAAGAAAAAGCCTGGCAAATGAATATAGGGTGATCATTACTGATGTTTTGGGTGGTGATATCCA
ATTTGACTTCTACCACCGCTGGTTTGATGCTATATTTGTGGCTAGTGCGTTCCTGTCCTTGCTTCTGATTTCTGCCCAATACACCACC
AGGCAAACAGACAAGCATCCGATTGATTGA

>SC2-Amino Acid Sequences
MGWGTVVYEGAVVGSSLVGLGWAGLWFLNRRLYKEYEERRVLVQILFGLVFAFSCNLFELVLFEILPVLSKHARFLNWHLDLFCLILL
LVFVLPYYHCYLLLRNSGVRRERSCLVAALFLLVFLYGFWRMGIHFPMPSPEKGFFTMPQLVSRIGVIGVSVMAVLSGFGAVNLPYSYL
SLFIREIDETDIKTLERQLMQSMETCTAKKKKIILSQMEMERIQGSEEKLKARSFLKRIVGTVVRSVQEDQTEQDIKNLEAEVQALEELSK
QLFLEIYELRQAKIAAAYSRTWRGHLQNLLGYALSVYCVYKMLKSLQSVVFKESGSVDPVTMTITIFLRHFDIGIDAALLSQYISLMFIG
MLVVISVRGFLANVMKFFFAVSRVGSGSTTNVVLFLSEIMGMYFISSILLIRKSLANEYRVIITDVLGGDIQFDFYHRWFDAIFVASAFLSL
LLISAQYTTRQTDKHPID*

>SC3-CDS
ATGGGGTGGGGCACAGTGGTTTACGAGGGCGCGGTCGTCGGCTCGTCGCTGGTGGGGCTGGGCTGGGCGGGGCTGTGGTTCCTGA
ACCGGCGGCTGTACAAGGAGTACGAGGAGCGGCGGGTGCTGGTGCAGATCCTCTTCGGCCTCGTCTTCGCCTTCTCCTGCAACCTC
TTCGAGCTCGTTCTCTTCGAGATCCTCCCCGTCCTCTCCAAGCATGCGCGCTTCCTCAACTGGCACCTCGACCTCTTCTGCCTCATC
CTCCTCCTCGTCTTCGTGCTCCCCTACTACCACTGCTATCTTCTGCTCCGTAACTCAGGGGTGAGGAGGGAGCGGTCGTGCCTCGTC
GCGGCGCTCTTTCTGCTGGTCTTCCTATACGGGTTCTGGCGCATGGGGATTCACTTCCCCATGCCTTCACCAGAGAAGGGTTTTTTT
ACGATGCCGCAGTTGGTCAGTAGGATTGGGGTGATTGGAGTGAGTGTCATGGCTGTTCTTTCTGGTTTTGGTGCTGTCAATCTGCC
ATACAGTTATCTGTCACTCTTCATCAGGGAAATTGATGAAACAGACATCAAAACCTTGGAACGGCAGCTGATGCAATCCATGGAG
ACATGTACTGCTAAGAAGAAGAAAATTATTTTGTCCCAGATGGAGATGGAGAGGATTCAAGGATCAGAGGAGAAGCTAAAGGCC
AGATCGTTTCTGAAGCGTATAGTGGGAACTGTTGTCAGATCTGTGCAGGAAGATCAAACTGAGCAGGATATAAAAAACTTAGAAG
CAGAAGTCCAGGCACTGGAAGAGCTTTCCAAACAGCTGTTCCTTGAGATATATGAACTCCGTCAGGCTAAGATAGCTGCTGCGTA
TTCTCGAACGTGGAGAGGGCATCTTCAGAATCTACTTGGATATGCTTTGTCGGTGTATTGTGTTTATAAGATGCTCAAGTCCTTGC
AGAGTGTAGTCTTTAAGGAGTCAGGCTCTGTTGATCCTGTAACAATGACAATAACGATTTTCCTGAGACATTTTGACATTGGCATT
GATGCTGCACTGTTATCTCAGTATATATCTTTGATGTTCATTGGGATGTTGGTTGTCATATCTGTTCGAGGTTTCTTGGCTAATGTT
ATGAAGTTCTTCTTCGCTGTTTCTAGAGTTGGGAGTGGGTCGACAACTAATGTTGTCCTTTTCCTATCAGAGATCATGGGCATGTAC
TTCATATCTTCCATTCTTCTTATAAGAAAAAGCCTGGCAAATGAATATAGGGTGATCATTACTGATGTTTTGGGTGGTGATATCCA
ATTTGACTTCTACCACCGCTGGTTTGATGCTATATTTGTGGCTAGTGCGTTCCTGTCCTTGCTTCTGATTTCTGCCCAATACACCACC
AGGCAAACAGACAAGCATCCGATTGATTGA

>SC3-Amino Acid Sequences
MGWGTVVYEGAVVGSSLVGLGWAGLWFLNRRLYKEYEERRVLVQILFGLVFAFSCNLFELVLFEILPVLSKHARFLNWHLDLFCLILL
LVFVLPYYHCYLLLRNSGVRRERSCLVAALFLLVFLYGFWRMGIHFPMPSPEKGFFTMPQLVSRIGVIGVSVMAVLSGFGAVNLPYSYL
SLFIREIDETDIKTLERQLMQSMETCTAKKKKIILSQMEMERIQGSEEKLKARSFLKRIVGTVVRSVQEDQTEQDIKNLEAEVQALEELSK
QLFLEIYELRQAKIAAAYSRTWRGHLQNLLGYALSVYCVYKMLKSLQSVVFKESGSVDPVTMTITIFLRHFDIGIDAALLSQYISLMFIG
MLVVISVRGFLANVMKFFFAVSRVGSGSTTNVVLFLSEIMGMYFISSILLIRKSLANEYRVIITDVLGGDIQFDFYHRWFDAIFVASAFLSL
LLISAQYTTRQTDKHPID*

>SC4-CDS
ATGGGGTGGGGCACAGTGGTTTACGAGGGCGCGGTCGTCGGCTCGTCGCTGGTGGGGCTGGGCTGGGCGGGGCTGTGGTTCCTGA
ACCGGCGGCTGTACAAGGAGTACGAGGAGCGGCGGGTGCTGGTGCAGATCCTCTTCGGCCTCGTCTTCGCCTTCTCCTGCAACCTC
TTCGAGCTCGTTCTCTTCGAGATCCTCCCCGTCCTCTCCAAGCATGCGCGCTTCCTCAACTGGCACCTCGACCTCTTCTGCCTCATC
CTCCTCCTCGTCTTCGTGCTCCCCTACTACCACTGCTATCTTCTGCTCCGTAACTCAGGGGTGAGGAGGGAGCGGTCGTGCCTCGTC
GCGGCGCTCTTTCTGCTGGTCTTCCTATACGGGTTCTGGCGCATGGGGATTCACTTCCCCATGCCTTCACCAGAGAAGGGTTTTTTT
ACGATGCCGCAGTTGGTCAGTAGGATTGGGGTGATTGGAGTGAGTGTCATGGCTGTTCTTTCTGGTTTTGGTGCTGTCAATCTGCC
ATACAGTTATCTGTCACTCTTCATCAGGGAAATTGATGAAACAGACATCAAAACCTTGGAACGGCAGCTGATGCAATCCATGGAG
ACATGTACTGCTAAGAAGAAGAAAATTATTTTGTCCCAGATGGAGATGGAGAGGATTCAAGGATCAGAGGAGAAGCTAAAGGCC
AGATCGTTTCTGAAGCGTATAGTGGGAACTGTTGTCAGATCTGTGCAGGAAGATCAAACTGAGCAGGATATAAAAAACTTAGAAG
CAGAAGTCCAGGCACTGGAAGAGCTTTCCAAACAGCTGTTCCTTGAGATATATGAACTCCGTCAGGCTAAGATAGCTGCTGCGTA
TTCTCGAACGTGGAGAGGGCATCTTCAGAATCTACTTGGATATGCTTTGTCGGTGTATTGTGTTTATAAGATGCTCAAGTCCTTGC
AGAGTGTAGTCTTTAAGGAGGCTCTGTTGATCCTGTAACAATGACAATAACGATTTTCCTGAGACATTTTGACATTGGCATTGATG
TTGCACTGTTATCTCAGTATATATCTTTGATGTTCATTGGGATGTTGGTTGTCATATCTGTTCGAGGTTTCTTGGCTAATGTTATGAA
GTTCTTCTTCGCTGTTTCTAGAGTTGGGAGTGGGTCGACAACTAATGTTGTCCTTTTCCTATCAGAGATCATGGGCATGTACTTCAT
ATCTTCCATTCTTCTTATAAGAAAAAGCCTGGCAAATGAATATAGGGTGATCATTACTGATGTTTTGGGTGGTGATATCCAATTTG
ACTTCTACCACCGCTGGTTTGATGCTATATTTGTGGCTAGTGCGTTCCTGTCCTTGCTTCTGATTTCTGCCCAATACACCACCAGGC
AAACAGACAAGCATCCGATTGATTGA

>SC4-Amino Acid Sequences
MGWGTVVYEGAVVGSSLVGLGWAGLWFLNRRLYKEYEERRVLVQILFGLVFAFSCNLFELVLFEILPVLSKHARFLNWHLDLFCLILL
LVFVLPYYHCYLLLRNSGVRRERSCLVAALFLLVFLYGFWRMGIHFPMPSPEKGFFTMPQLVSRIGVIGVSVMAVLSGFGAVNLPYSYL
SLFIREIDETDIKTLERQLMQSMETCTAKKKKIILSQMEMERIQGSEEKLKARSFLKRIVGTVVRSVQEDQTEQDIKNLEAEVQALEELSK
QLFLEIYELRQAKIAAAYSRTWRGHLQNLLGYALSVYCVYKMLKSLQSVVFKESGSVDPVTMTITIFLRHFDIGIDAALLSQYISLMFIG
MLVVISVRGFLANVMKFFFAVSRVGSGSTTNVVLFLSEIMGMYFISSILLIRKSLANEYRVIITDVLGGDIQFDFYHRWFDAIFVASAFLSL
LLISAQYTTRQTDKHPID*

>SC5-CDS
ATGGGGTGGGGCACAGTGGTTTACGAGGGCGCGGTCGTCGGCTCGTCCCTGGTGGGGCTGGGCTGGGCGGGGCTGTGGTTCCTGA
ACCGGCGGCTGTACAAGGAGTACGAGGAGCGGCGGGTGCTGGTGCAGATCCTCTTCGGCCTCGTCTTCGCCTTCTCCTGCAACCTC
TTCGAGCTCGTTCTCTTCGAGATCCTCCCCGTCCTCTCCAAGCATGCGCGCTTCCTCAACTGGCACCTCGACCTCTTCTGCCTCATC
CTCCTCCTCGTCTTCGTGCTCCCCTACTACCACTGCTATCTTCTGCTCCGTAACTCAGGGGTGAGGAGGGAGCGGTCGTGCCTCGTC
GCGGCGCTCTTTCTGCTGGTCTTCCTATACGGGTTCTGGCGCATGGGGATTCACTTCCCCATGCCTTCACCAGAGAAGGGTTTTTTT
ACGATGCCGCAGTTGGTCAGTAGGATTGGGGTGATTGGAGTGAGTGTCATGGCTGTTCTTTCTGGTTTTGGTGCTGTCAATCTGCC
ATACAGTTATCTGTCACTCTTCATCAGGGAAATTGATGAAACAGACATCAAAACCTTGGAACGGCAGCTGATGCAATCCATGGAG
ACATGTACTGCTAAGAAGAAGAAAATTATTTTGTCCCAGATGGAGATGGAGAGGATTCAAGGATCAGAGGAGAAGCTAAAGGCC
AGATCGTTTCTGAAGCGTATAGTGGGAACTGTTGTCAGATCTGTGCAGGAAGATCAAACTGAGCAGGATATAAAAAACTTAGAAG
CAGAAGTCCAGGCACTGGAAGAGCTTTCCAAACAGCTGTTCCTTGAGATATATGAACTACGTCAGGCTAAGATAGCTGCTGCGTA
TTCTCGAACGTGGAGAGGGCATCTTCAGAATCTACTTGGATATGCTTTGTCGGTGTATTGTGTTTATAAGATGCTCAAGTCCTTGC
AGAGTGTAGTCTTTAAGGAGTCAGGCTCTGTTGATCCTGTAACAATGACAATAACGATTTTCCTGAGACATTTTGACATTGGCATT
GATGCTGCACTGTTATCTCAGTATATATCTTTGATGTTCATTGGGATGTTGGTTGTCATATCTGTTCGAGGTTTCTTGGCTAATGTT
ATGAAGTTCTTCTTCGCTGTTTCTAGAGTTGGGAGTGGGTCGACAACTAATGTTGTCCTTTTCCTATCAGAGATCATGGGCATGTAC
TTCATATCTTCCATTCTTCTTATAAGAAAAAGCCTGGCAAATGAATATAGGGTGATCATTACTGATGTTTTGGGTGGTGATATCCA
ATTTGACTTCTACCACCGCTGGTTTGATGCTATATTTGTGGCTAGTGCGTTCCTGTCCTTGCTTCTGATTTCTGCCCAATACACCACC
AGGCAAACAGACAAGCATCCGATTGATTGA

>SC5-Amino Acid Sequences
MGWGTVVYEGAVVGSSLVGLGWAGLWFLNRRLYKEYEERRVLVQILFGLVFAFSCNLFELVLFEILPVLSKHARFLNWHLDLFCLILL
LVFVLPYYHCYLLLRNSGVRRERSCLVAALFLLVFLYGFWRMGIHFPMPSPEKGFFTMPQLVSRIGVIGVSVMAVLSGFGAVNLPYSYL
SLFIREIDETDIKTLERQLMQSMETCTAKKKKIILSQMEMERIQGSEEKLKARSFLKRIVGTVVRSVQEDQTEQDIKNLEAEVQALEELSK
QLFLEIYELRQAKIAAAYSRTWRGHLQNLLGYALSVYCVYKMLKSLQSVVFKEALLIL-Q-Q-RFS-DILTLALMLHCYLSIYL*

>EC1-CDS
ATGGGGTGGGGCACAGTGGTTTACGAGGGCGCGGTCGTCGGCTCGTCGCTGGTGGGGCTGGGCTGGGCGGGGCTGTGGTTCCTGA
ACCGGCGGCTGTACAAGGAGTACGAGGAGCGGCGGGTGCTGGTGCAGATCCTCTTCGGCCTCGTCTTCGCCTTCTCCTGCAACCTC
TTCGAGCTCGTTCTCTTCGAGATCCTCCCCGTCCTCTCCAAGCATGCGCGCTTCCTCAACTGGCACGTCGACCTCTTCTGCCTCATC
CTCCTCCTCGTCTTCGTGCTCCCCTACTACCACTGCTATCTTCTGCTCCGTAACTCAGGAGTGAGGAGGGAGCGGTCGTGCCTCGTC
GCGGCGCTCTTTCTGCTGGTCTTCCTATACGGGTTCTGGCGCATGGGGATTCACTTCCCCATGCCTTCACCAGAGAAGGGTTTTTTT
ACGATGCCGCAGTTGGTCAGTAGGATTGGGGTGATTGGAGTGAGTGTCATGGCTGTTCTTTCTGGTTTTGGTGCTGTCAATCTGCC
ATACAGTTATCTGTCACTCTTCATCAGGGAAATTGATGAAACAGACATCAAAACCTTGGAACGGCAGCTGATGCAATCCATGGAG
ACATGTACTGCTAAGAAGAAGAAAATTATTTTGTCCCAGATGGAGATGGAGAGGATTCAAGGATCAGAGGAGAAGCTAAAGGCC
AGATCATTTCTGAAGCGTATAGTGGGAACTGTTGTCAGATCTGTGCAGGAAGATCAAACTGAGCAGGATATAAAAAACTTAGAAG
CAGAAGTCCAGGCACTGGAAGAGCTTTCCAAACAGCTGTTCCTTGAGATATATGAACTCCGTCAGGCTAAGATAGCTGCTGCATA
TTCTCGAACGTGGAGAGGGCATCTTCAGAATCTACTTGGATATGCTTTGTCGGTGTATTGTGTTTATAAGATGCTCAAGTCCTTGC
AGAGTGTAGTCTTTAAGGAGTCAGGCTCTGTTGATCCTGTAACAATGACAATAACGATTTTCCTGAGACATTTTGACATTGGCATT
GATGTTGCACTGTTATCTCAGTATATATCTTTGATGTTCATTGGGATGTTGGTTGTCATATCTGTTCGAGGTTTCTTGGCTAATGTTA
TGAAGTTCTTCTTCGCTGTTTCTAGAGTTGGGAGTGGGTCGACAACCAATGTTGTCCTTTTCCTATCAGAGATCATGGGCATGTACT
TCATATCTTCCATTCTTCTTATAAGAAAAAGCCTGGCAAATGAATATAGGGTGATCATTACTGATGTTTTGGGTGGTGATATCCAA
TTTGACTTCTACCACCGCTGGTTTGATGCTATATTTGTGGCTAGTGCGTTCCTGTCCTTGCTTCTGATTTCTGCCCAATACACCACCA
GGCAAACAGACAAGCATCCGATTGATTGA

>EC1-Amino Acid Sequences
MGWGTVVYEGAVVGSSLVGLGWAGLWFLNRRLYKEYEERRVLVQILFGLVFAFSCNLFELVLFEILPVLSKHARFLNWHVDLFCLILL
LVFVLPYYHCYLLLRNSGVRRERSCLVAALFLLVFLYGFWRMGIHFPMPSPEKGFFTMPQLVSRIGVIGVSVMAVLSGFGAVNLPYSYL
SLFIREIDETDIKTLERQLMQSMETCTAKKKKIILSQMEMERIQGSEEKLKARSFLKRIVGTVVRSVQEDQTEQDIKNLEAEVQALEELSK
QLFLEIYELRQAKIAAAYSRTWRGHLQNLLGYALSVYCVYKMLKSLQSVVFKESGSVDPVTMTITIFLRHFDIGIDVALLSQYISLMFIG
MLVVISVRGFLANVMKFFFAVSRVGSGSTTNVVLFLSEIMGMYFISSILLIRKSLANEYRVIITDVLGGDIQFDFYHRWFDAIFVASAFLSL
LLISAQYTTRQTDKHPID*

>EC2-CDS
ATGGGGTGGGGCACAGTGGTTTACGAGGGCGCGGTCGTCGGCTCGTCGCTGGTGGGGCTGGGCTGGGCGGGGCTGTGGTTCCTGA
ACCGGCGGCTGTACAAGGAGTACGAGGAGCGGCGGGTGCTGGTGCAGATCCTCTTCGGCCTCGTCTTCGCCTTCTCCTGCAACCTC
TTCGAGCTCGTTCTCTTCGAGATCCTCCCCGTCCTCTCCAAGCATGCGCGCTTCCTCAACTGGCACCTCGACCTCTTCTGCCTCATC
CTCCTCCTCGTCTTCGTGCTCCCCTACTACCACTGCTATCTTCTGCTCCGTAACTCAGGGGTGAGGAGGGAGCGGTCGTGCCTCGTC
GCGGCGCTCTTTCTGCTGGTCTTCCTATACGGGTTCTGGCGCATGGGGATTCACTTCCCCATGCCTTCACCAGAGAAGGGTTTTTTT
ACGATGCCGCAGTTGGTCAGTAGGATTGGGGTGATTGGAGTGAGTGTCATGGCTGTTCTTTCTGGTTTTGGTGCTGTCAATCTGCC
ATACAGTTATCTGTCACTCTTCATCAGGGAAATTGATGAAACAGACATCAAAACCTTGGAACGGCAGCTGATGCAATCCATGGAG
ACATGTACTGCTAAGAAGAAGAAAATTATTTTGTCCCAGATGGAGATGGAGAGGATTCAAGGATCAGAGGAGAAGCTAAAGGCC

AGATCGTTTCTGAAGCGTATAGTGGGAACTGTTGTCAGATCTGTGCAGGAAGATCAAACTGAGCAGGATATAAAAAACTTAGAAG
CAGAAGTCCAGGCACTAGAAGAGCTTTCCAAACAGCTGTTCCTTGAGATATATGAACTCCGTCAGGCTAAGATAGCTGCTGCGTA
TTCTCGAACGTGGAGAGGGCATCTTCAGAATCTACTTGGATATGCTTTGTCGGTGTATTGTGTTTATAAGATGCTCAAGTCCTTGC
AGAGTGTAGTCTTTAAGGAGTCAGGCTCTGTTGATCCTGTAACAATGACAATAACGATTTTCCTGAGACATTTTGACATTGGCATT
GATGTTGCACTGTTATCTCAGTATATATCTTTGATGTTCATTGGGATGTTGGTTGTCATATCTGTTCGAGGTTTCTTGGCTAATGTTA
TGAAGTTCTTCTTCGCTGTTTCTAGAGTTGGGAGTGGGTCGACAACCAATGTTGTCCTTTTCCTATCAGAGATCATGGGCATGTACT
TCATATCTTCCATTCTTCTTATAAGAAAAAGCCTGGCAAATGAATATAGGGTGATCATTACTGATGTTTTGGGTGGTGATATCCAA
TTTGACTTCTACCACCGCTGGTTTGATGCTATATTTGTGGCTAGTGCGTTCCTGTCCTTGCTTCTGATTTCTGCCCAATACACCACCA
GGCAAACAGACAAGCATCCGATTGATTGA

>EC2- Amino Acid Sequences
MGWGTVVYEGAVVGSSLVGLGWAGLWFLNRRLYKEYEERRVLVQILFGLVFAFSCNLFELVLFEILPVLSKHARFLNWHLDLFCLILL
LVFVLPYYHCYLLLRNSGVRRERSCLVAALFLLVFLYGFWRMGIHFPMPSPEKGFFTMPQLVSRIGVIGVSVMAVLSGFGAVNLPYSYL
SLFIREIDETDIKTLERQLMQSMETCTAKKKKIILSQMEMERIQGSEEKLKARSFLKRIVGTVVRSVQEDQTEQDIKNLEAEVQALEELSK
QLFLEIYELRQAKIAAAYSRTWRGHLQNLLGYALSVYCVYKMLKSLQSVVFKESGSVDPVTMTITIFLRHFDIGIDVALLSQYISLMFIG
MLVVISVRGFLANVMKFFFAVSRVGSGSTTNVVLFLSEIMGMYFISSILLIRKSLANEYRVIITDVLGGDIQFDFYHRWFDAIFVASAFLSL
LLISAQYTTRQTDKHPID*

>EC3-CDS
ATGGGGTGGGGCACAGTGGTTTACGAGGGCGCGGTCGTCGGCTCGTCCCTGGTGGGGCTGGGCTGGGCGGGGCTGTGGTTCCTGA
ACCGGCGGCTGTACAAGGAGTACGAGGAGCGGCGGGTGCTGGTGCAGATCCTCTTCGGCCTCGTCTTCGCCTTCTCCTGCAACCTC
TTCGAGCTCGTTCTCTTCGAGATCCTCCCCGTCCTCTCCAAGCATGCGCGCTTCCTCAACTGGCACCTCGACCTCTTCTGCCTCATC
CTCCTCCTCGTCTTCGTGCTCCCCTACTACCACTGCTATCTTCTGCTCCGTAACTCAGGGGTGAGGAGGGAGCGGTCGTGCCTCGTC
GCGGCGCTCTTTCTGCTGGTCTTCCTATACGGGTTCTGGCGCATGGGGATTCACTTCCCCATGCCTTCACCAGAGAAGGGTTTTTTT
ACGATGCCGCAGTTGGTCAGTAGGATTGGGGTGATTGGAGTGAGTGTCATGGCTGTTCTTTCTGGTTTTGGTGCTGTCAATCTGCC
ATACAGTTATCTGTCACTCTTCATCAGGGAAATTGATGAAACAGACATCAAAACCTTGGAACGGCAGCTGATGCAATCCATGGAG
ACATGTACTGCTAAGAAGAAGAAAATTATTTTGTCCCAGATGGAGATGGAGAGGATTCAAGGATCAGAGGAGAAGCTAAAGGCC
AGATCGTTTCTGAAGCGTATAGTGGGAACTGTTGTCAGATCTGTGCAGGAAGATCAAACTGAGCAGGATATAAAAAACTTAGAAG
CAGAAGTCCAGGCACTGGAAGAGCTTTCCAAACAGCTGTTCCTTGAGATATATGAACTACGTCAGGCTAAGATAGCTGCTGCGTA
TTCTCGAACGTGGAGAGGGCATCTTCAGAATCTACTTGGATATGCTTTGTCGGTGTATTGTGTTTATAAGATGCTCAAGTCCTTGC
AGAGTGTAGTCTTTAAGGAGTCAGGCTCTGTTGATCCTGTAACAATGACAATAACGATTTTCCTGAGACATTTTGACATTGGCATT
GATGTTGCACTGTTATCTCAGTATATATCTTTGATGTTCATTGGGATGTTGGTTGTCATATCTGTTCGAGGTTTCTTGGCTAATGTTA
TGAAGTTCTTCTTCGCCGTTTCTAGAGTTGGGAGTGGGTCAACAACCAATGTTGTCCTTTTCCTATCAGAGATCATGGGCATGTACT
TCATATCTTCCATTCTTCTTATAAGAAAAAGCCTGGCAAATGAATATAGGGTGATCATTACTGATGTTTTGGGTGGTGATATCCAA
TTTGACTTCTACCACCGCTGGTTTGATGCTATATTTGTGGCTAGTGCGTTCCTGTCCTTGCTTCTGATTTCTGCCCAATACACCACCA
GGCAAACAGACAAGCATCCGATTGATTGA

>EC3- Amino Acid Sequences
MGWGTVVYEGAVVGSSLVGLGWAGLWFLNRRLYKEYEERRVLVQILFGLVFAFSCNLFELVLFEILPVLSKHARFLNWHLDLFCLILL
LVFVLPYYHCYLLLRNSGVRRERSCLVAALFLLVFLYGFWRMGIHFPMPSPEKGFFTMPQLVSRIGVIGVSVMAVLSGFGAVNLPYSYL
SLFIREIDETDIKTLERQLMQSMETCTAKKKKIILSQMEMERIQGSEEKLKARSFLKRIVGTVVRSVQEDQTEQDIKNLEAEVQALEELSK
QLFLEIYELRQAKIAAAYSRTWRGHLQNLLGYALSVYCVYKMLKSLQSVVFKESGSVDPVTMTITIFLRHFDIGIDVALLSQYISLMFIG
MLVVISVRGFLANVMKFFFAVSRVGSGSTTNVVLFLSEIMGMYFISSILLIRKSLANEYRVIITDVLGGDIQFDFYHRWFDAIFVASAFLSL
LLISAQYTTRQTDKHPID*

>EC4-CDS
ATGGGGTGGGGCACAGTGGTTTACGAGGGCGCGGTCGTCGGCTCGTCGCTGGTGGGGCTGGGCTGGGCGGGGCTGTGGTTCCTGA
ACCGGCGGCTGTACAAGGAGTACGAGGAGCGGCGGGTGCTGGTGCAGATCCTCTTCGGCCTCGTCTTCGCCTTCTCCTGCAACCTC
TTCGAGCTCGTTCTCTTCGAGATCCTCCCCGTCCTCTCCAAGCATGCGCGCTTCCTCAACTGGCACCTCGACCTCTTCTGCCTCATC
CTCCTCCTCGTCTTCGTGCTCCCCTACTACCACTGCTATCTTCTGCTCCGTAACTCAGGGGTGAGGAGGGAGCGGTCGTGCCTCGTC
GCGGCGCTCTTTCTGCTGGTCTTCCTATACGGGTTCTGGCGCATGGGGATTCACTTCCCCATGCCTTCACCAGAGAAGGGTTTTTTT
ACGATGCCGCAGTTGGTCAGTAGGATTGGGGTGATTGGAGTGAGTGTCATGGCTGTTCTTTCTGGTTTTGGTGCTGTCAATCTGCC
ATACAGTTATCTGTCACTCTTCATCAGGGAAATTGATGAAACAGACATCAAAACCTTGGAACGGCAGCTGATGCAATCCATGGAG
ACATGTACTGCTAAGAAGAAGAAAATTATTTTGTCCCAGATGGAGATGGAGAGGATTCAAGGATCAGAGGAGAAGCTAAAGGCC
AGATCGTTTCTGAAGCGTATAGTGGGAACTGTTGTCAGATCTGTGCAGGAAGATCAAACTGAGCAGGATATAAAAAACTTAGAAG
CAGAAGTCCAGGCACTGGAAGAGCTTTCCAAACAGCTGTTCCTTGAGATATATGAACTCCGTCAGGCTAAGATAGCTGCTGCGTA
TTCTCGAACGTGGAGAGGGCATCTTCAGAATCTACTTGGATATGCTTTGTCGGTGTATTGTGTTTATAAGATGCTCAAGTCCTTGC
AGAGTGTAGTCTTTAAGGAGTCAGGCTCTGTTGATCCTGTAACAATGACAATAACGATTTTCCTGAGACATTTTGACATTGGCATT
GATGTTGCACTGTTATCTCAGTATATATCTTTGATGTTCATTGGGATGTTGGTTGTCATATCTGTTCGAGGTTTCTTGGCTAATGTTA
TGAAGTTCTTCTTCGCTGTTTCTAGAGTTGGGAGTGGGTCGACAACTAATGTTGTCCTTTTCCTATCAGAGATCATGGGCATGTACT
TCATATCTTCCATTCTTCTTATAAGAAAAAGCCTGGCAAATGAATATAGGGTGATCATTACTGATGTTTTGGGTGGTGATATCCAA

TTTGACTTCTACCACCGCTGGTTTGATGCTATATTTGTGGCTAGTGCGTTCCTGTCCTTGCTTCTGATTTCTGCCCAATACACCACCA
GGCAAACAGACAAGCATCCGATTGATTGA

>EC4- Amino Acid Sequences
MGWGTVVYEGAVVGSSLVGLGWAGLWFLNRRLYKEYEERRVLVQILFGLVFAFSCNLFELVLFEILPVLSKHARFLNWHLDLFCLILL
LVFVLPYYHCYLLLRNSGVRRERSCLVAALFLLVFLYGFWRMGIHFPMPSPEKGFFTMPQLVSRIGVIGVSVMAVLSGFGAVNLPYSYL
SLFIREIDETDIKTLERQLMQSMETCTAKKKKIILSQMEMERIQGSEEKLKARSFLKRIVGTVVRSVQEDQTEQDIKNLEAEVQALEELSK
QLFLEIYELRQAKIAAAYSRTWRGHLQNLLGYALSVYCVYKMLKSLQSVVFKESGSVDPVTMTITIFLRHFDIGIDVALLSQYISLMFIG
MLVVISVRGFLANVMKFFFAVSRVGSGSTTNVVLFLSEIMGMYFISSILLIRKSLANEYRVIITDVLGGDIQFDFYHRWFDAIFVASAFLSL
LLISAQYTTRQTDKHPID*

>EC5-CDS
ATGGGGTGGGGCACAGTGGTTTACGAGGGCGCGGTCGTCGGCTCGTCGCTGGTGGGGCTGGGCTGGGCGGGGCTGTGGTTCCTGA
ACCGGCGGCTGTACAAGGAGTACGAGGAGCGGCGGGTGCTGGTGCAGATCCTCTTCGGCCTCGTCTTCGCCTTCTCCTGCAACCTC
TTCGAGCTCGTTCTCTTCGAGATCCTCCCCGTCCTCTCCAAGCATGCGCGCTTCCTCAACTGGCACGTCGACCTCTTCTGCCTCATC
CTCCTCCTCGTCTTCGTGCTCCCCTACTACCACTGCTATCTTCTGCTCCGTAACTCAGGAGTGAGGAGGGAGCGGTCGTGCCTCGTC
GCGGCGCTCTTTCTGCTGGTCTTCCTATACGGGTTCTGGCGCATGGGGATTCACTTCCCCATGCCTTCACCAGAGAAGGGTTTTTTT
ACGATGCCGCAGTTGGTCAGTAGGATTGGGGTGATTGGAGTGAGTGTCATGGCTGTTCTTTCTGGTTTTGGTGCTGTCAATCTGCC
ATACAGTTATCTGTCACTCTTCATCAGGGAAATTGATGAAACAGACATCAAAACCTTGGAACGGCAGCTGATGCAATCCATGGAG
ACATGTACTGCTAAGAAGAAGAAAATTATTTTGTCCCAGATGGAGATGGAGAGGATTCAAGGATCAGAGGAGAAGCTAAAGGCC
AGATCGTTTCTGAAGCGTATAGTGGGAACTGTTGTCAGATCTGTGCAGGAAGATCAAACTGAGCAGGATATAAAAAACTTAGAAG
CAGAAGTCCAGGCACTAGAAGAGCTTTCCAAACAGCTGTTCCTTGAGATATATGAACTCCGTCAGGCTAAGATAGCTGCTGCGTA
TTCTCGAACGTGGAGAGGGCATCTTCAGAATCTACTTGGATATGCTTTGTCGGTGTATTGTGTTTATAAGATGCTCAAGTCCTTGC
AGAGTGTAGTCTTTAAGGAGTCAGGCTCTGTTGATCCTGTAACAATGACAATAACGATTTTCCTGAGACATTTTGACATTGGCATT
GATGTTGCACTGTTATCTCAGTATATATCTTTGATGTTCATTGGGATGTTGGTTGTCATATCTGTTCGAGGTTTCTTGGCTAATGTTA
TGAAGTTCTTCTTCGCTGTTTCTAGAGTTGGGAGTGGGTCGACAACCAATGTTGTCCTTTTCCTATCAGAGATCATGGGCATGTACT
TCATATCTTCCATTCTTCTTATAAGAAAAAGCCTGGCAAATGAATATAGGGTGATCATTACTGATGTTTTGGGTGGTGATATCCAA
TTTGACTTCTACCACCGCTGGTTTGATGCTATATTTGTGGCTAGTGCGTTCCTGTCCTTGCTTCTGATTTCTGCCCAATACACCACCA
GGCAAACAGACAAGCATCCGATTGATTGA

>EC5- Amino Acid Sequences

MGWGTVVYEGAVVGSSLVGLGWAGLWFLNRRLYKEYEERRVLVQILFGLVFAFSCNLFELVLFEILPVLSKHARFLNWHVDLFCLILL
LVFVLPYYHCYLLLRNSGVRRERSCLVAALFLLVFLYGFWRMGIHFPMPSPEKGFFTMPQLVSRIGVIGVSVMAVLSGFGAVNLPYSYL
SLFIREIDETDIKTLERQLMQSMETCTAKKKKIILSQMEMERIQGSEEKLKARSFLKRIVGTVVRSVQEDQTEQDIKNLEAEVQALEELSK
QLFLEIYELRQAKIAAAYSRTWRGHLQNLLGYALSVYCVYKMLKSLQSVVFKESGSVDPVTMTITIFLRHFDIGIDVALLSQYISLMFIG
MLVVISVRGFLANVMKFFFAVSRVGSGSTTNVVLFLSEIMGMYFISSILLIRKSLANEYRVIITDVLGGDIQFDFYHRWFDAIFVASAFLSL
LLISAQYTTRQTDKHPID*

Supplementary Dataset. Nucleotide and amino acid sequences of ten GPCR homeoalleles cloned and sequenced from sugarcane (SC) and energy cane (EC) varieties.
